# Mitigation mechanism of silicon and iron co-application to cadmium toxicity in tomato seedlings by integrated transcriptomic and physiological correlation analysis

**DOI:** 10.3389/fpls.2025.1555618

**Published:** 2025-06-30

**Authors:** Xiaoting Zhou, Wenjie Wang, Deyang Ye, Xiaoru Liu, Chutong Peng, Yunxin Tang, Lihong Su, Shaobo Cheng, Kai Cao, Qiyuan Lei, Tonghua Pan, Zhongqun He

**Affiliations:** 1 College of Horticulture, Sichuan Agricultural University, Chengdu, Sichuan, China; 2 The Agriculture Ministry Key Laboratory of Agricultural Engineering in the Middle and Lower Reaches of Yangtze River, Institute of Agricultural Facilities and Equipment, Jiangsu Academy of Agricultural Sciences, Nanjing, Jiangsu, China; 3 School of Agricultural Engineering, Jiangsu University, Zhenjiang, Jiangsu, China

**Keywords:** cadmium, iron, silicon, tomato seedlings, toxicity

## Abstract

Cadmium (Cd) readily accumulates in plants during agricultural activities, leading to diminished crop yields and quality and posing a risk to humans. Silicon (Si) and iron (Fe) have shown promise in mitigating Cd toxicity, but the efficacy of their combined application to alleviate the stress of Cd remains unclear. This study focused on the physiological and transcriptomic responses of tomato seedlings to Cd stress. When tomato seedlings were subjected to Cd stress, the application of external Si and Fe effectively alleviated Cd toxicity; increased photosynthetic pigment content, antioxidant enzyme activity, and transcription factor; and improved plant growth. Transcriptome analyses revealed that photosynthetic antenna proteins, photosynthesis-related processes, and glutathione metabolism were significantly overrepresented among differentially expressed genes when plants were subjected to Si-Fe competition under Cd stress conditions. Fructose-bisphosphate aldolase is a critical component in the mechanism of the effectiveness of Si-Fe in mitigating Cd toxicity. The hub genes CAB13 and CAB6A could potentially play a role in the modulation of photorespiration and light-capture processes when subjected to Si-Fe treatment. These findings offer novel insights into the environmental impacts and underlying mechanisms governing the roles of Si and Fe in reducing Cd toxicity.

## 1 Introduction

Cadmium negatively affects plant and animal growth, and is a substantial global environmental contaminant (Rai et al., 2019). The manifestations of Cd toxicity in plants include inhibitions in growth, modifications in photosynthetic processes, alterations in enzyme activity, disruptions in protein metabolism, and changes in membrane functions (Zhang et al., 2024). Moreover, the Cd taken up by plants can traverse the food chain, potentially compromising food safety and posing risks to animal and human health (Chunhabundit, 2016; Han et al., 2016). Hence, it is crucial to ensure human safety and facilitate normal crop development by decreasing Cd toxicity and limiting its movement and accumulation in plants. Various approaches have been suggested to mitigate the phytotoxic effects in Cd-affected regions, including agronomic management practices, bioremediation methodologies, and molecular biology strategies (Zhang et al., 2019; Li et al., 2019; Hu et al., 2019; Tang et al., 2017). Recently, nutrient management has emerged as a favored approach, as it not only fosters robust crop development but also effectively curtails Cd accumulation in consumable portions. Reducing Cd accumulation in plants can be reduced by managing the interactions between Cd and other nutrients (Zhu et al., 2023; Wang et al., 2019).

Multiple studies have demonstrated that Si mitigates damage caused by Cd in plant cells (Zhao et al., 2022). Moreover, Si enhances the capacity to mitigate Cd bioavailability, creates a protective barrier within the root architecture, and modulates the subcellular distribution of Cd by influencing the expression of genes involved in Cd-uptake transport (Ma et al., 2015; Vaculik et al., 2012; Wu et al., 2016; Greger et al., 2016). Moreover, the majority of the Cd that remains in the cell wall is found within pectin, and the incorporation of Si enhances the capacity of both pectin and hemicellulose to adsorb Cd (Wei et al., 2021). Additionally, foliar application of Si might also help plants mitigate the harmful effects of Cd toxicity and accumulation by binding to Cd within the aerial cell walls. For example, applying Si to rich leaves helps alleviate Cd toxicity by decreasing the accumulation and redistribution of Cd in the aboveground plant components (Wang et al., 2015).

Iron is a crucial component of many iron-binding proteins and plays a multifaceted role in various plant biological processes (Zhu et al., 2023; Gao and Dubos, 2021). Moreover, Fe is the most effective micronutrient for mitigating Cd toxicity in plants (Emamverdian et al., 2015), and enhancing its supply mitigated the effects of cadmium toxicity, as evidenced by the development of larger leaves and a notable increase in chlorophyll content (Shao et al., 2008; Liu et al., 2017).

Tomato (*Solanum lycopersicon* L.), a crucial vegetable crop (Li et al., 2018), is extensively cultivated and consumed worldwide. China is one of the leading producers and consumers of tomatoes globally, and tomato production serves as a pivotal strategy for farmers to enhance their earnings and contribute to foreign exchange revenue (Xu et al., 2000). Concurrently, tomato is frequently utilized as an important research subject with considerable research importance across various scientific domains, including genetics, cell biology, bioengineering, molecular biology, and genomics (The Tomato Genome Consortium, 2019; Sun et al., 2018). However, tomatoes are sensitive to Cd (Haider et al., 2021), and escalating Cd pollution is a growing concern as it diminishes the yield and quality of tomatoes and other vegetables and also jeopardizes human health (Xu et al., 2020; Zhang et al., 2018).

Numerous studies have explored the interactions between Si and other mineral elements to mitigate Cd toxicity. For example, B and Si have been shown to decrease Cd uptake and transport in rice by suppressing the expression of Cd transporter genes, enhancing antioxidant enzyme activities, alleviating Cd-induced oxidative stress, and reducing Cd accumulation and toxicity (Chen et al., 2019). Moreover, the findings revealed that both Si and Se mitigated Cd toxicity. The concurrent application of Si and Se facilitated the growth of rice plants, diminished malondialdehyde (MDA) levels in both the root system and aboveground parts, and decreased the Cd transport factor (Huang et al., 2021). Furthermore, the concurrent application of Si and salicylic acid was more effective at mitigating the adverse effects of Cd on maize seedlings (Singh et al., 2019).

However, the function of Si-Fe co-application in mitigating Cd toxicity in tomato remains unclear. Therefore, to investigate the interactions between Si, Fe, and Cd, we studied the growth of tomato seedlings under the interaction of Si, Fe, and Cd. Furthermore, we revealed the molecular-level differences of key Cd transporter genes by qRT-PCR analysis and transcriptome analysis to provide a theoretical basis for the prevention and control of soil pollution in vegetables and the safe production of tomatoes.

## 2 Materials and methods

### 2.1 Plant materials and treatment designs

The experiment was conducted from October 2022 to November 2023 in the plant factory and laboratory at the College of Horticulture, Sichuan Agricultural University (plant factories provide artificially regulated environments for plant growth needs) (Hu et al., 2014; Zou et al., 2024). Tomatoes (*Lycopersicon c.v.* “Lv Feicui”) were used as the treatment material. The seeds were previously screened by the laboratory and sourced from Shou Guang Hongwei Seed Industry Co., Shandong Province (Su et al., 2021). The hydroponic nutrient solution used for testing was 1/2 Hoagland nutrient solution (pH 6.2), and the stress treatments were designed by augmenting this base solution with 50 μmol·L^−1^ CdCl_2_·2.5H_2_O, 1.5 mmol·L^−1^ Na_2_SiO_3_·9H_2_O, and 100 μmol·L^−1^ EDTA-Fe. Silicon and iron concentrations were rigorously quantified in preliminary analyses. Owing to the absence of the silicon efflux transporter protein (SILsi-2-L) in the tomato root system, silicon predominantly accumulates within the roots, exhibiting limited translocation to the shoots; thus, foliar application is employed to ensure efficient silicon delivery to aerial tissues. Details of the experimental treatments are listed in **Table 1**.

**Table 1:** Nutrient solution for each treatment.

Treatment group	Disposition
CK	/
Cd	Addition to 50 μmol·L^−1^ Cd stress treatment
CF	Addition to 50 μmol·L^−1^ Cd stress treatment + 100 μmol·L^−1^ EDTA-Fe treatment (root application)
CS	Addition to 50 μmol·L^−1^ Cd stress treatment + 1.5 mmol·L^−1^ Si treatment (leaf application)
CSF	Addition to 50 μmol·L^−1^ Cd stress treatment + 1.5 mmol·L^−1^ Si treatment (leaf application) + 100 μmol·L^−1^ EDTA-Fe treatment (root application)

1/2 Hoagland nutrient solution was added in every treatment.

### 2.2 Preliminary treatment

Seeds were soaked in warm broth and placed in a germination chamber maintained at 30°C for 3 to 5 days. Once the seedlings reached 5 mm in length, they were transplanted into 72-hole trays and transferred to an incubator set to 26°C, with relative humidity controlled between 60% and 80%, and a light intensity of 250 μmol·m^-2^·s^-1^. When the tomato plants developed four fully expanded true leaves, uniform and vigorous seedlings were selected and transplanted into 12-hole hydroponic boxes measuring 38 × 28 × 14 cm. The experiment was conducted using a randomized design with five distinct treatments.

### 2.3 Determination of biomass indexes

Tomato seedlings underwent hydroponic treatment for 7 days. Ten sample plants were randomly selected for each treatment. The height of the plants was measured with a straightedge after absorbing water with blotting paper. Fresh and dry weights were measured as described by Zhou et al. (2023).

### 2.4 Determination of Cd content

Tomato seedlings were subjected to hydroponic treatment for 7 days. Subsequently, their roots were immersed in a 20 mmol/L Na_2_-EDTA solution for 20 min to effectively remove metal chelates. After rinsing thoroughly with deionized water, plants from each treatment were carefully separated into aboveground and root tissues, then dried at 75°C. For sample preparation, 0.2 g of the dried tissue was placed into a microwave digestion vessel containing 5 mL of concentrated nitric acid (HNO_3_) and left to pre-digest overnight. The following day, 2 mL of hydrogen peroxide (H_2_O_2_) was added to complete the digestion process. Once cooled to room temperature, the digest was evaporated to near dryness, diluted to 25 mL with 1% (v/v) HNO_3_ (analytical grade), and filtered. Cadmium concentrations in roots and leaves were then quantified using atomic absorption spectrophotometry (Soylak et al., 2007).

### 2.5 Determination of photosynthesis traits and chlorophyll level

Tomato seedlings were grown hydroponically for 7 days. For chlorophyll fluorescence measurements, the second fully expanded leaf from the apex was selected after complete dark adaptation and analyzed using a PAM-2500 chlorophyll fluorometer (Walz, Germany). Gas exchange parameters were measured with an LI-6400 portable photosynthesis system (Li-COR, USA), providing accurate assessment of leaf physiological responses. To quantify chlorophyll content, 0.2 g of leaf tissue was weighed and immersed in 25 mL of 95% ethanol, sealed, and incubated in darkness within a foam box for 24 to 36 h until the leaves were fully bleached. Absorbance readings were taken at 470, 645, and 663 nm, with 95% ethanol serving as the blank reference, following the method of Zhou et al. (2021). This integrated methodology allowed for precise evaluation of photosynthetic efficiency and pigment concentration under hydroponic conditions.

### 2.6 Assessment of antioxidant enzyme activity and MDA levels

Tomato seedlings underwent hydroponic treatment for 7 days. The MDA content was quantified using the thiobarbituric acid method of extraction (TBA) (Senthilkumar et al., 2021). The SOD activity was quantified using the nitrotetrazolium blue chloride (NBT) technique (Cheng et al., 2015), POD activity was evaluated using the guaiacol procedure (Mika and Lüthje, 2003), and CAT activity was determined using the UV absorption method (Zeng et al., 2019).

### 2.7 Transcriptome analysis

Tomato seedlings underwent hydroponic treatment for 7 days. The blades were ground into powder with liquid nitrogen. Total RNA was extracted using The TRIzol reagent (Invitrogen, Carlsbad, CA, USA). Total RNA was detected using the Agilent 2100 Bioanalyzer (Thermo Scientific, Waltham, MA, USA). Construction and sequencing of the RNA SEQ libraries were performed by Novozymes Biotech Co., Ltd. (Beijing, China). DESeq2 software (1.20.0) was used to analyze the differential expression of the two comparison combinations, with a *p*-value of < 0.05 and |log2FoldChange| > 0. Gene Ontology (GO) and Kyoto Encyclopedia of Genes and Genomes (KEGG) pathway enrichment were assessed using cluster Profiler software version 3.4.4. Weighted Gene Co-expression Network Analysis (WGCNA) was used to construct co-expression network and hub genes (Langfelder and Horvath, 2008), and Cytoscape3.7.2 software constructed the network.

### 2.8 Gene expression analysis for transcriptomic data

Tomato seedlings underwent hydroponic treatment for 7 days. The tomato seedlings were left in liquid nitrogen, with three replications per treatment and 0.3 g of leaves was clipped each time. Subsequently, RNA was isolated and converted into cDNA by reverse transcription (Zhou et al., 2023) (accession number: PRJNA1245030). The cDNA was then subjected to real-time fluorescence expression analysis of the relevant genes. Primers were synthesized by bioengineering, and the details are listed in **Supplementary Table S1**.

Real-time quantitative fluorescence analysis was conducted in a 20-μL reaction volume comprising 1 μL of template cDNA, 0.2 μL of each of the upstream and downstream primers, 10 μL of SYBR Green PCR Master Mix (Bao Bioengineering Co., Ltd., China), and the balance was made up with 20 μL of double-distilled water. The relative quantification of gene expression was counted using 2^−ΔΔCt^.

Using the Bio-Rad CFX96TM real-time PCR system (Bio-Rad, Hercules, CA, USA), the PCR conditions were as follows: pre-denaturation at 95°C for 1 min, denaturation at 95°C for 10 s for 40 cycles, and annealing and extension at 58°C for 15-s cycles. Melting curves were generated to verify the specificity of the primer amplification.

### 2.9 Data processing and correlation analysis

The dataset was meticulously organized using Microsoft Excel 2016. Comprehensive statistical analyses—including analysis of variance (ANOVA), Shapiro–Wilk normality assessments, Duncan’s multiple range tests, and correlation analyses—were executed with SPSS software. High-quality data visualization and graphical mapping were accomplished using GraphPad Prism 8.

## 3 Results

### 3.1 Changes in plant growth and Cd content

**Figure 1:**
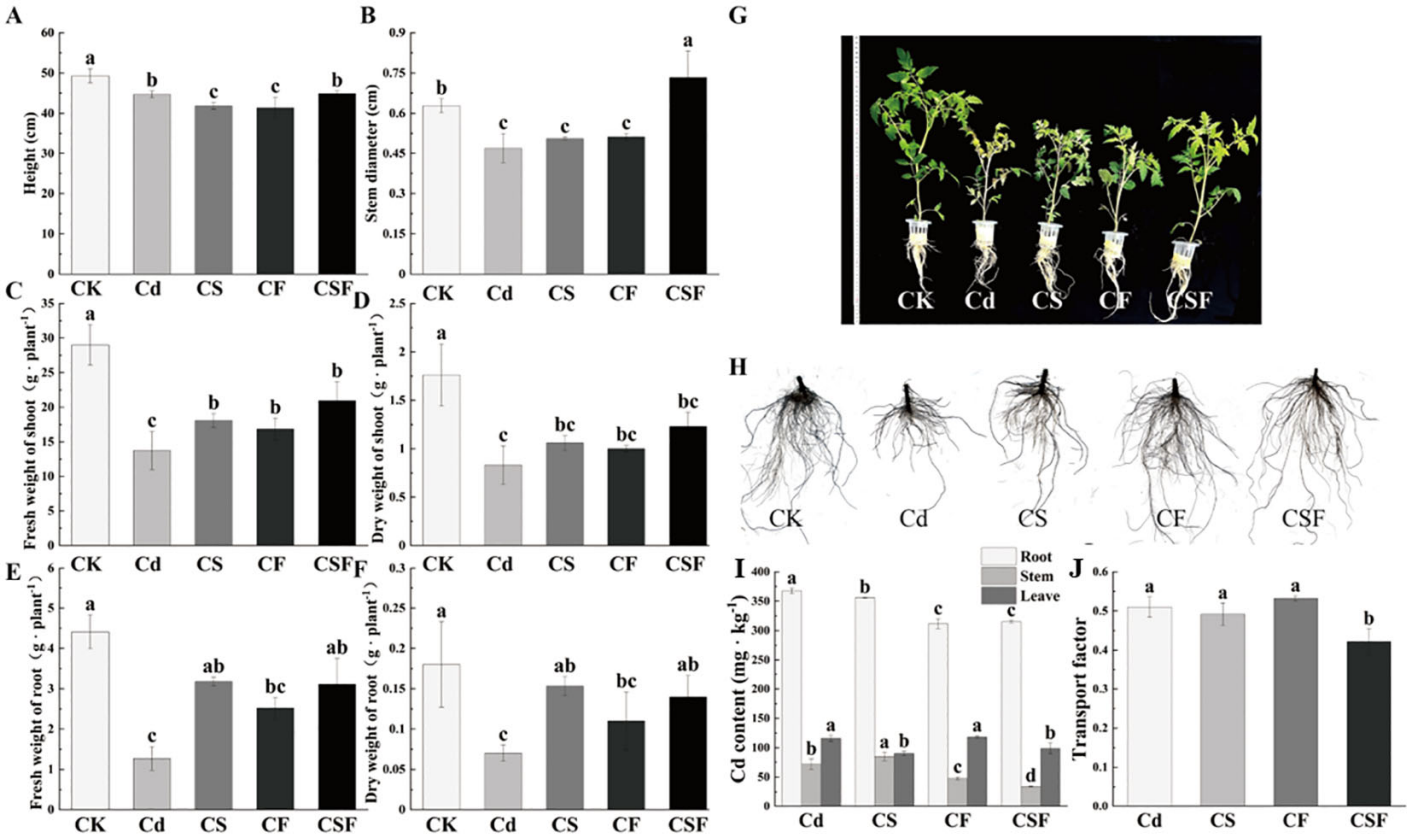
Different treatments on growth indicators and Cd content in tomato seedlings. CF: Cd stress and EDTA-Fe treatment (root application). CS: Cd stress and Si treatment (leaf application). CSF: Cd stress, EDTA-Fe treatment (root application) and Si treatment (leaf application). **(A)** Plant height, **(B)** stem diameter, **(C)** fresh weight of shoot, **(D)** dry weight of shoot, **(E)** fresh weight of root, **(F)** dry weight of shoot, **(G)** plant phenotypes, **(H)** plant root, **(I)** Cd content, and **(J)** transport factor. Different letters (a–c) on the bar plots indicate significant difference at *p* < 0.05 using S-W normal distribution (*p* > 0.05), one-way analysis of variance with Duncan’s multiple-range test.

The co-application of silicon (Si) and iron (Fe) markedly enhanced the biomass of tomato seedlings subjected to cadmium (Cd) stress (**Figures 1A–F**). Compared to the control, Cd exposure significantly inhibited seedling growth; however, supplementation with Si and Fe individually promoted growth, with their combined application (CSF) producing the most substantial improvements. Specifically, CSF increased stem diameter, shoot fresh weight, and root fresh weight by 64.46%, 52.56%, and 145.14%, respectively. Parallel increases in shoot and root dry weights further corroborate the alleviation of Cd-induced growth inhibition by CSF. Morphological assessments depicted in **Figures 1G, H** visually confirm these enhancements, underscoring the efficacy of Si and Fe co-application in mitigating Cd stress effects on tomato seedlings.

**Figures 1I, J** illustrate the impact of various treatments on cadmium (Cd) distribution within tomato seedlings and the associated transport factor. Treatments with silicon, iron, and their combined application (CSF) significantly reduced Cd accumulation in roots by 3.16%, 15.19%, and 14.29%, respectively, compared to the Cd-only group. Notably, CSF also markedly decreased Cd concentrations in stems and leaves. These results indicate that the synergistic application of Si and Fe effectively inhibits both the uptake and translocation of Cd in tomato seedlings, thereby mitigating Cd accumulation in aerial tissues.

### 3.2 Chlorophyll fluorescence parameters and antioxidant enzyme activity

**Figure 2:**
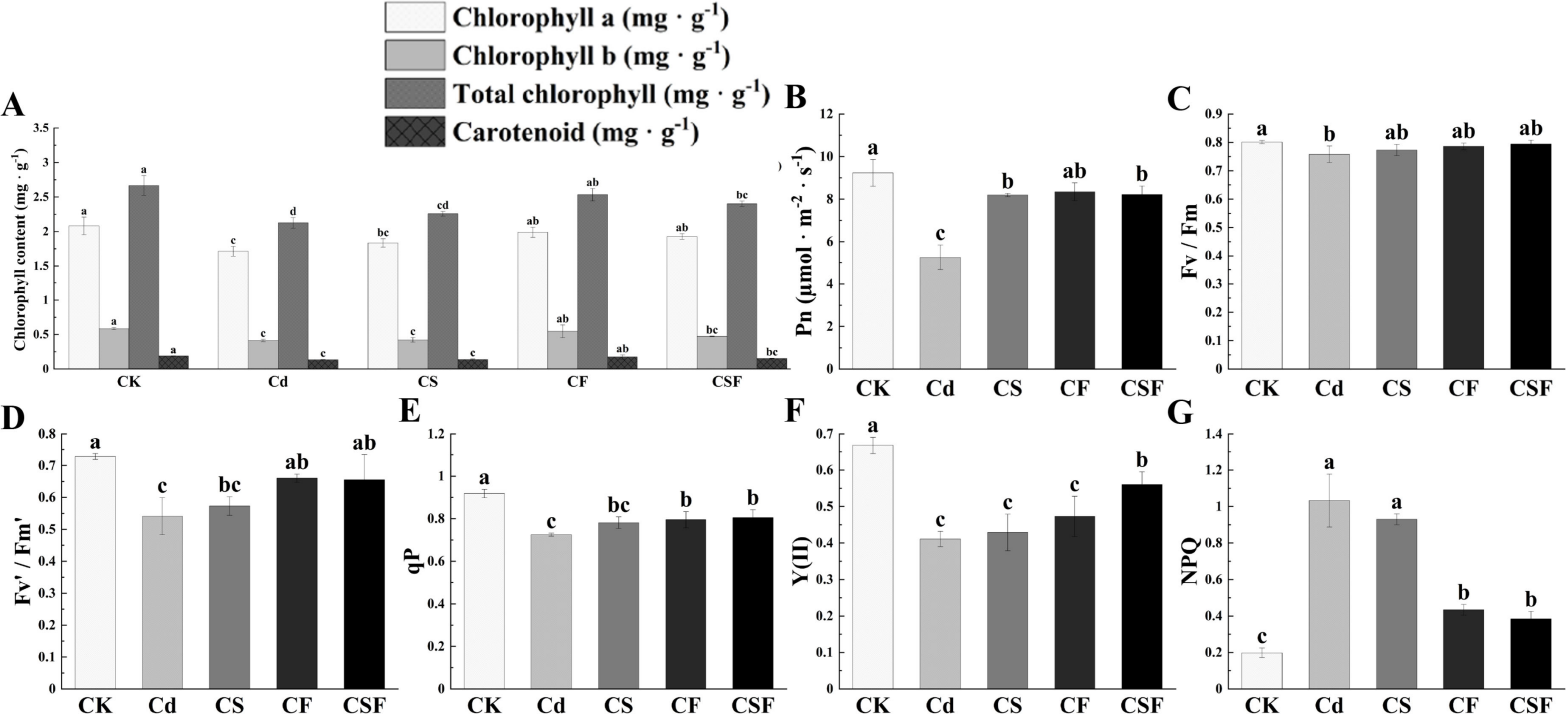
Different treatments on chlorophyll levels and chlorophyll fluorescence parameters in tomato seedlings. **(A)** Chlorophyll content, **(B)** net photosynthetic rate (Pn), **(C)** maximum quantum yield of PSII (Fv/Fm), **(D)** effective quantum yield of PSII (Fv′/Fm′), **(E)** photochemical quenching coefficient (qP), **(F)** actual photosynthetic efficiency of photosystem II [Y(II)], and **(G)** non-photochemical quenching coefficient (NPQ). Different letters (a–c) on the bar plots indicate significant difference at *p* < 0.05 using S-W normal distribution (*p* > 0.05), one-way analysis of variance with Duncan’s multiple-range test.

Compared to the control (CK), cadmium (Cd) exposure pronouncedly suppressed the biosynthesis of photosynthetic pigments in tomato seedlings. While silicon supplementation alone (CS) elicited a modest, non-significant increase in pigment levels relative to Cd treatment, the combined application of silicon and iron (CSF) produced statistically significant enhancements of 12.6%, 14.6%, 12.9%, and 13.6% across the measured photosynthetic pigments, underscoring its pronounced efficacy in alleviating Cd-induced impairment of photosynthetic capacity (**Figure 2A**).

Compared with the control (CK), Cd stress reduced net photosynthetic rate (Pn), maximum quantum efficiency of PSII (Fv/Fm), effective quantum yield of PSII (Fv′/Fm′), actual photochemical efficiency [Y(II)], and photochemical quenching coefficient (qP), while increasing non-photochemical quenching (NPQ). The combined application of silicon and iron (CSF) significantly increased Pn and partially alleviated the decreases in Fv/Fm, Fv′/Fm′, Y(II), and qP, restoring Fv/Fm to control levels. Additionally, CSF increased Y(II) by 127.5% and reduced NPQ by 29.36% (**Figures 2B–G**). Cd treatment also decreased transpiration rate (Tr) and stomatal conductance (Gs), while elevating intercellular CO_2_ concentration (Ci) compared to CK. Exogenous application of Si, Fe, and their combination mitigated these effects on Tr, Ci, and Gs under Cd stress (**Supplementary Figure S1**). These results indicate that CSF effectively improves the photosynthetic performance of tomato seedlings under Cd stress.

Compared to the control (CK), cadmium (Cd) stress and treatments with silicon (CS) and iron (CF) induced significant alterations in the antioxidant defense system of tomato seedlings. Notably, the combined application of silicon and iron (CSF) elicited a pronounced upregulation of antioxidant enzyme activities, including superoxide dismutase (SOD), peroxidase (POD), and catalase (CAT), relative to Cd treatment alone. Cd exposure resulted in maximal accumulation of MDA, indicative of enhanced lipid peroxidation and membrane oxidative damage. However, CSF treatment significantly mitigated MDA accumulation, reflecting attenuation of oxidative membrane injury (**Supplementary Figure S2**).

### 3.3 Correlation analysis

**Figure 3:**
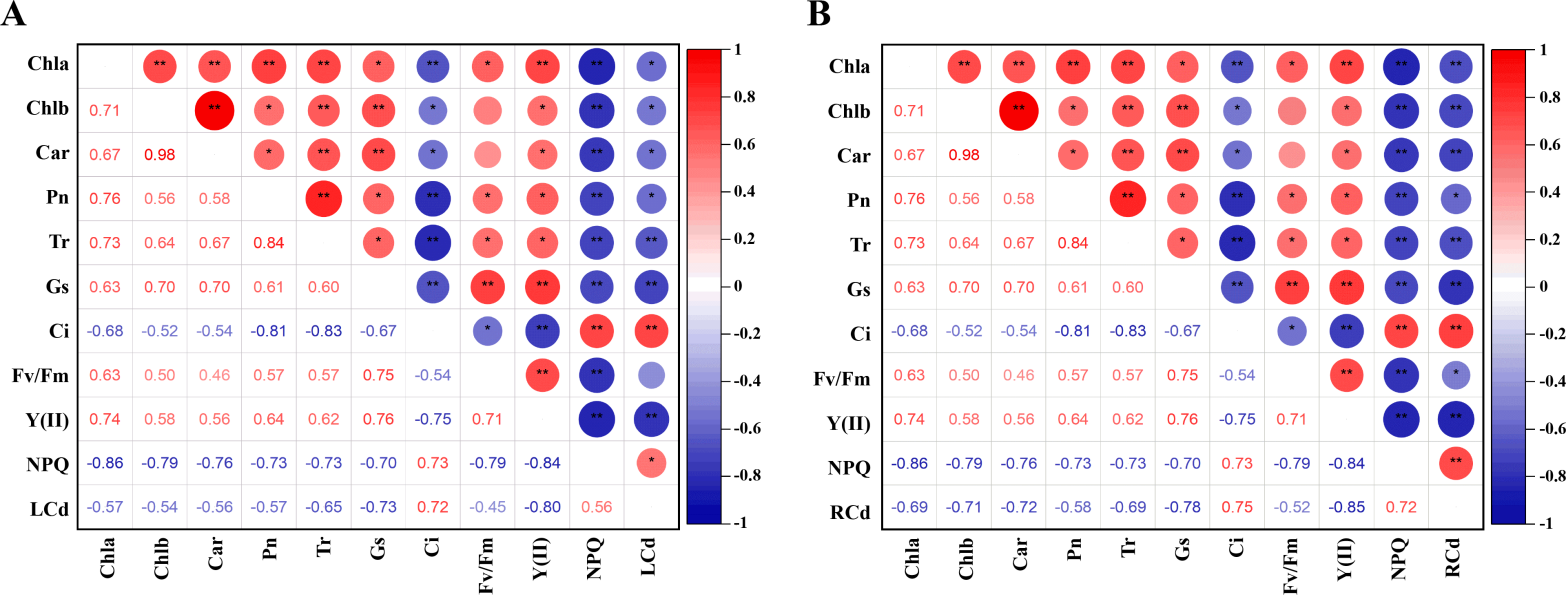
Different treatments on correlation analysis in tomato seedlings. **(A)** Correlation analysis of leaves, **(B)** Correlation analysis of root. The correlation analysis utilizes the Pearson correlation coefficient. Red and blue bubbles indicate positive and negative correlations (*p < 0.05, **p < 0.01).

We conducted correlation analyses to explore the relationship between the photosynthetic system and Cd content in tomato plants. As shown in **Figure 3**, leaf Cd content exhibited a significant positive correlation with non-photochemical quenching (NPQ) and intercellular CO_2_ concentration (Ci), while showing significant negative correlations with chlorophyll a (Chl a), chlorophyll b (Chl b), carotenoids (Car), and net photosynthetic rate (Pn). Moreover, leaf Cd content was highly negatively correlated with the effective quantum yield of photosystem II [Y(II)] and stomatal conductance (Gs). Similarly, root Cd content was strongly positively correlated with NPQ and Ci, significantly negatively correlated with Pn and the maximum quantum efficiency of PSII (Fv/Fm), and highly negatively correlated with Chl a, Chl b, Car, transpiration rate (Tr), Gs, and Y(II). These patterns clearly demonstrate that Cd accumulation inflicts severe impairment on the photosynthetic machinery of tomato seedlings, disrupting pigment levels, gas exchange, and photochemical efficiency, thereby compromising overall photosynthetic performance.

### 3.4 Transcriptomics analysis and RT-qPCR validation

To validate the accuracy and reproducibility of the RNA-seq data, 11 differentially expressed genes (DEGs) were randomly selected for fluorescence quantification via RT-qPCR. The expression patterns observed through RT-qPCR were consistent with the transcriptome analysis, confirming the reliability of the RNA-seq results (**Supplementary Figures S4** and **S7**). In addition, as shown in **Supplementary Figure S6**, the correlation coefficient (*r*²) of 0.8214 between RNA-seq and RT-qPCR data was significant, indicating that our RNA-seq data were reliable.

**Figure 4:**
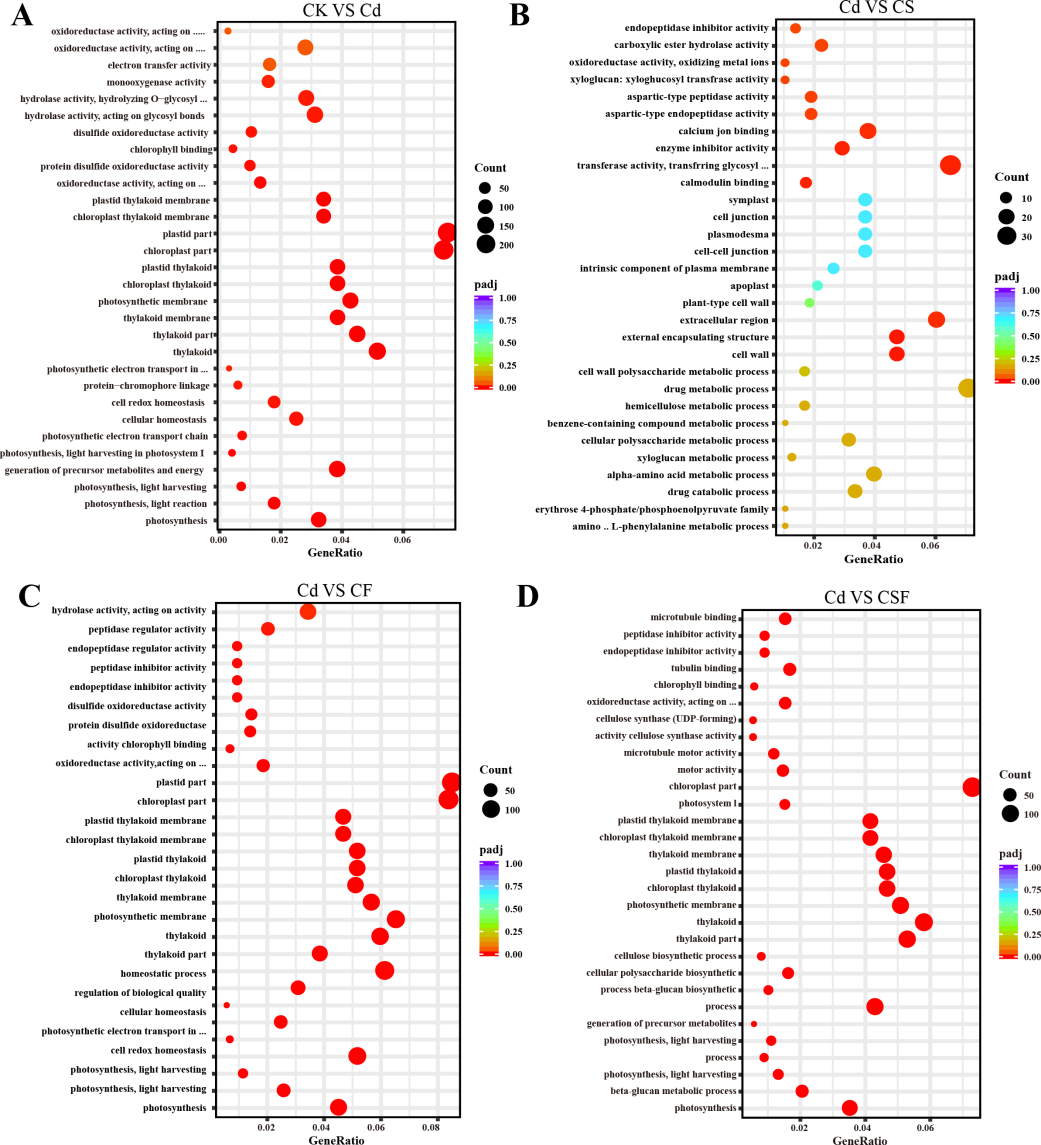
GO analysis in tomato seedling leaves. **(A)** CK vs. Cd, **(B)** Cd vs. CS, **(C)** Cd vs. CF, and **(D)** Cd vs. CSF. The size of the bubbles indicates the number and the color indicates the *p*-value: the redder the color, the lower the *p*-value.

The GO enrichment analysis revealed distinct functional overrepresentations across different treatment comparisons (**Figure 4**). When comparing the control with cadmium (Cd) stress, GO terms related to chloroplast and plastid components, thylakoid structures, photosynthesis, and oxidoreductase activity were significantly enriched, indicating perturbations in photosynthetic and redox processes. In the comparison between Cd and silicon treatment, the most significantly enriched GO categories included transferase activity, external cell regions, external encapsulation structures, and cell wall components, reflecting modifications in enzymatic functions and cell wall architecture. Furthermore, comparisons of Cd with iron treatment (CF) and Cd with combined silicon and iron treatment (CSF) consistently showed significant enrichment of GO terms associated with chloroplast, thylakoid, and photosynthesis, underscoring the restorative effects of these treatments on photosynthetic machinery.

**Figure 5:**
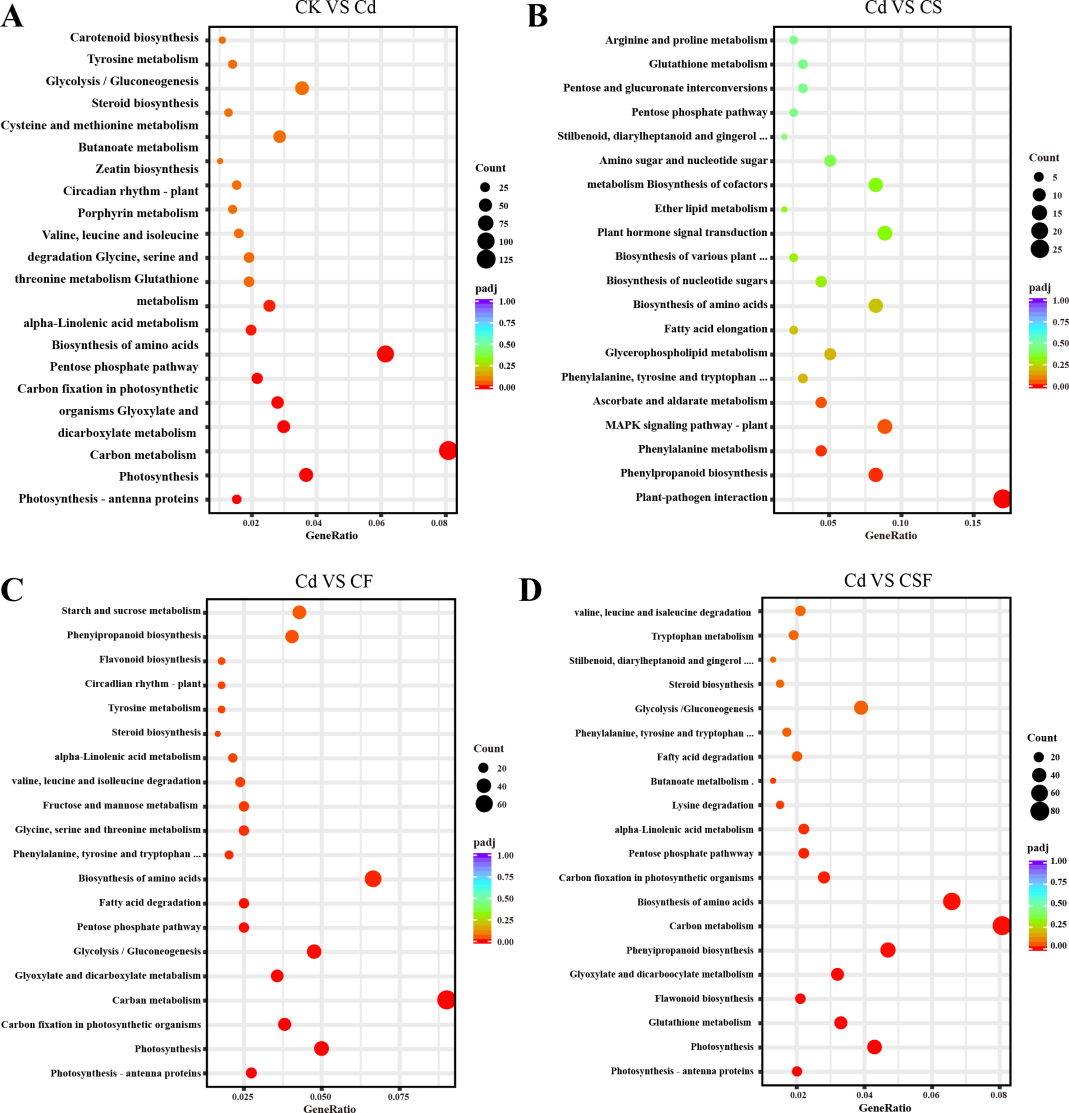
KEGG analysis in tomato seedling leaves. **(A)** CK vs. Cd, **(B)** Cd vs. CS, **(C)** Cd vs. CF, and **(D)** Cd vs. CSF. The size of the bubbles indicates the number, and the color indicates the *p*-value: the redder the color, the lower the *p*-value.

Pathway enrichment analysis revealed distinct metabolic alterations across treatments (**Figure 5**). The comparison between the control (CK) and cadmium (Cd) treatment identified carbon metabolism as the most significantly enriched pathway, involving 128 DEGs, with amino acid biosynthesis as the second most enriched pathway. In the comparison of Cd versus silicon treatment (CS), pathways associated with plant–pathogen interactions were significantly enriched, indicating activation of stress response mechanisms. Comparisons of Cd with iron treatment (CF) and with combined silicon and iron treatment (CSF) demonstrated significant enrichment in carbon metabolism, amino acid biosynthesis, and photosynthesis pathways. These results underscore the pivotal roles of primary metabolism and photosynthetic processes in mediating the plant’s response to Cd stress and its mitigation by Si and Fe treatments.

### 3.5 Common DEG analysis

Based on the analysis above, we determined that cadmium (Cd) significantly disrupts the photosynthetic and antioxidant systems in tomato seedlings. Accordingly, we focused on a set of common DEGs related to photosynthesis, antioxidant enzyme activity, and mechanisms involved in Cd detoxification.

#### 3.5.1 DEGs involving photosynthesis and carbon metabolism

**Figure 6:**
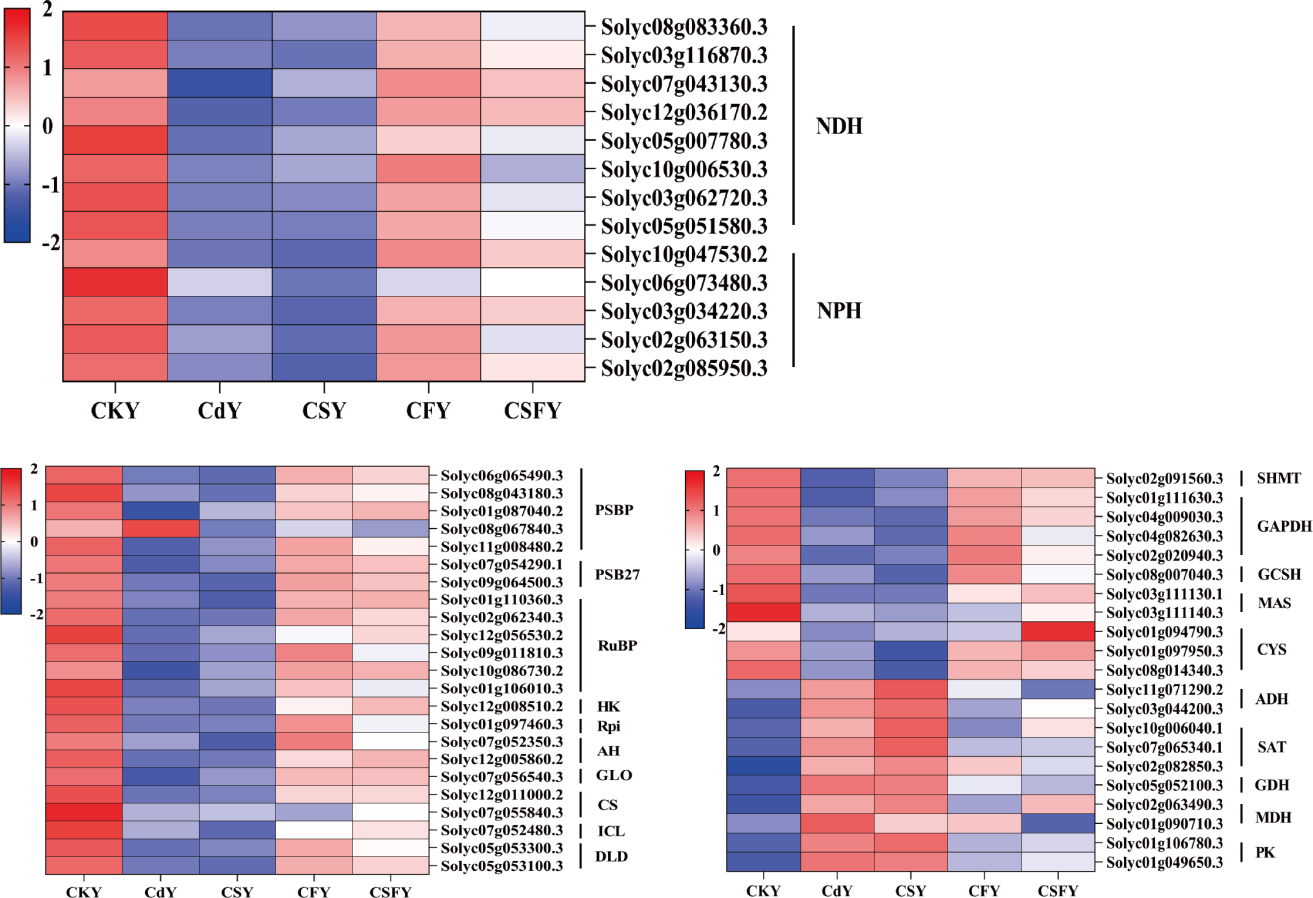
DEGs involving photosynthesis and carbon metabolism in tomato seedlings. Color indicates the expression level: the redder the color, the higher the number of DEGs.

Cadmium markedly alters the expression of genes involved in photosynthesis and carbon metabolism in tomato seedlings. Silicon alone exerts minimal mitigation of Cd effects, whereas iron (CF) and the combined silicon and iron treatment (CSF) significantly alleviate Cd-induced disruptions in these pathways (**Figure 6**).

#### 3.5.2 DEGs involving cell wall

**Figure 7:**
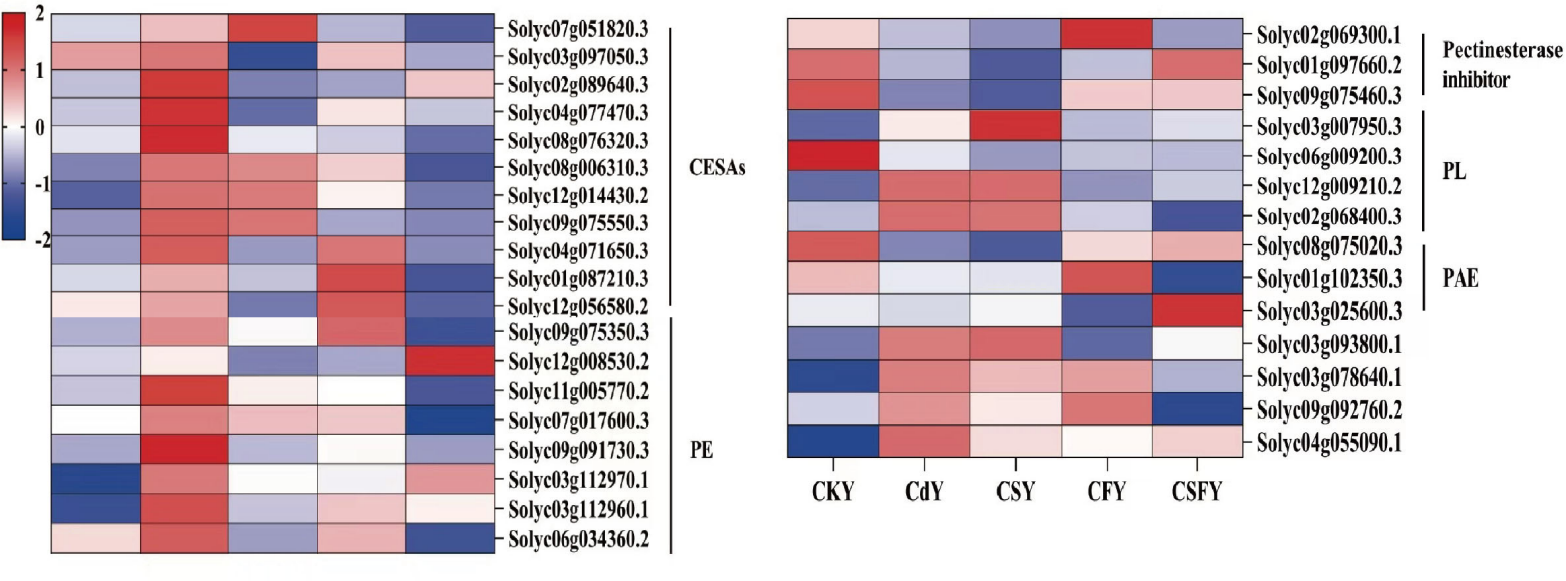
DEGs involved cell walls of tomato seedlings. Cellulose synthase (CESAs) and pectin acetyl esterase (PAE) favor cell wall synthesis. Pectin esterase (PE), pectate lyase (PL), and pectin esterase inhibitors favor pectin decomposition. Color indicates the expression level: the redder the color, the higher the number of DEGs.

The cell wall, characterized by its thick, resilient, and slightly elastic structure, acts as the primary barrier against Cd stress in plants (Cosgrove, 2023). Exposure to Cd upregulates genes involved in cell wall synthesis, such as CESAs, PE, and PL, while downregulating pectin esterase inhibitors and PAE. This response is partially mitigated by Si or Fe application, with their combined use exerting a markedly stronger restorative effect (**Figure 7**).

#### 3.5.3 DEGs involving antioxidant enzymes

**Figure 8:**
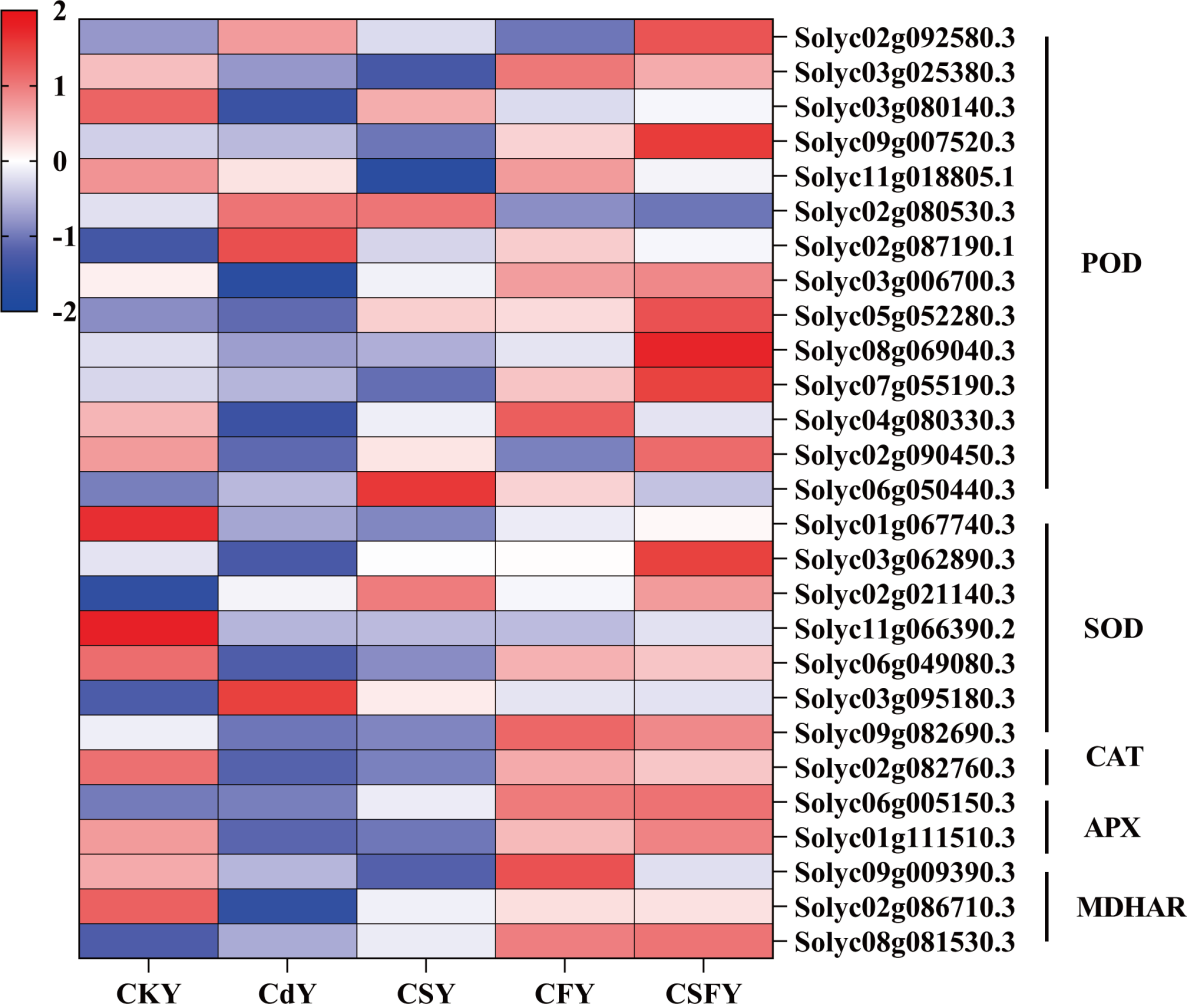
DEGs involved in antioxidant enzymes in tomato seedlings. Color indicates the expression level: the redder the color, the higher the number of DEGs.

DEGs related to antioxidant enzymes (POD, SOD, CAT, APX, and MDHAR) were significantly downregulated following Cd application. Conversely, in the presence of CS or CF, the expression of these genes was upregulated. The expression of genes encoding antioxidant enzymes was significantly upregulated in tomato seedlings when Si and Fe were co-applied. This suggests that Si and Fe can produce positive benefits for each other to alleviate the damage of tomato seedlings by Cd (**Figure 8**).

#### 3.5.4 DEGs involving glutathione

**Figure 9:**
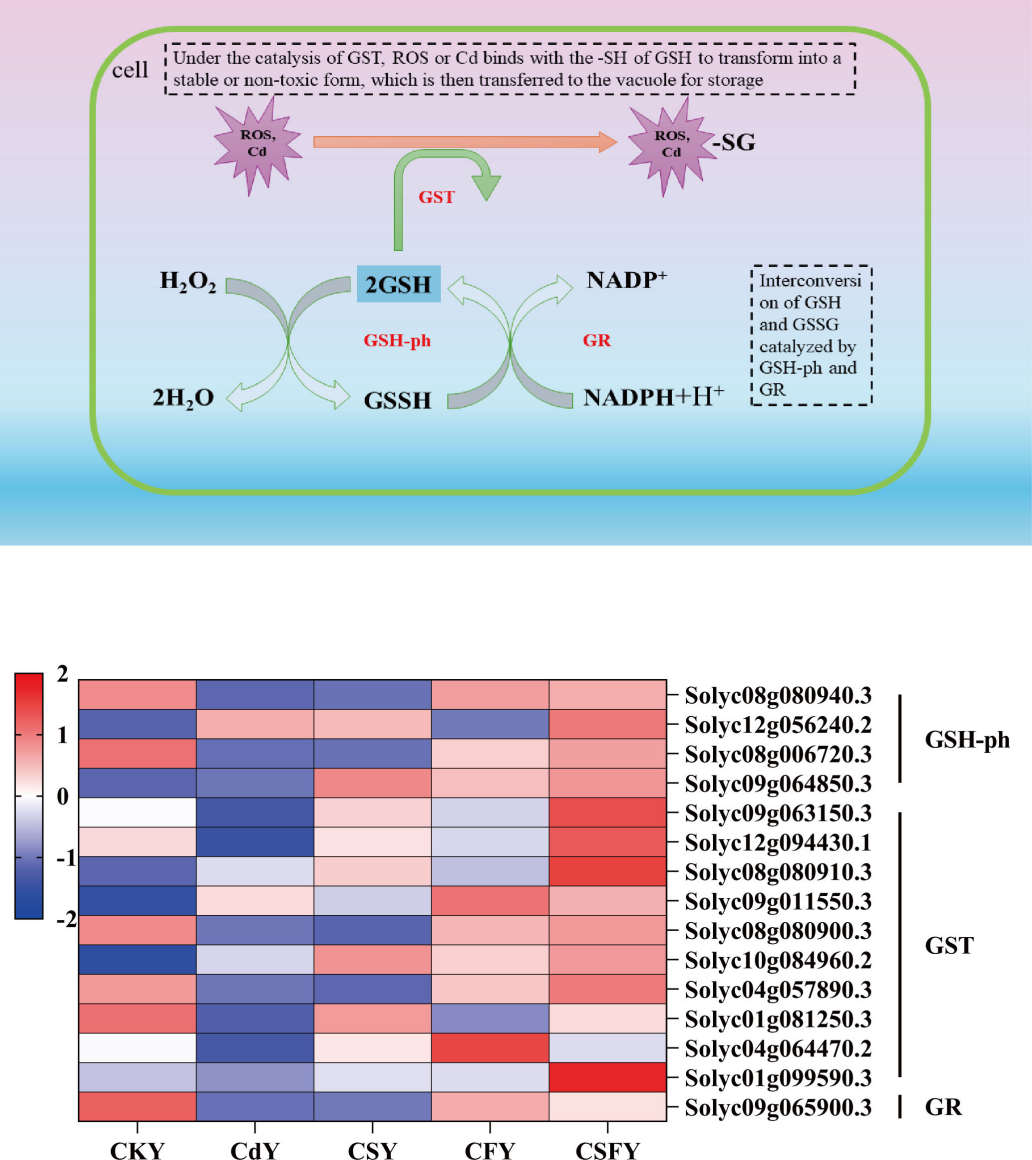
DEGs involved antioxidant enzymes in tomato seedlings. A shows a schematic diagram of GSH scavenging by ROS and Cd^2+^. Color of B indicates the expression level: the redder the color, the higher the number of DEGs.

Glutathione (GSH), an endogenous antioxidant, not only neutralizes free radicals in plants but also scavenges heavy metals, including Hg, Cd, and As (Hasanuzzaman et al., 2017). Here, Cd inhibited the expression of glutathione-ph, glutathione S-transferase (GST), and glutathione reductase (GR). Conversely, the expression of these genes showed an opposite trend in CS and CF compared to that in Cd and was significantly upregulated in CSF (**Figure 9**).

#### 3.5.5 DEGs involving element transporters

Elemental transporter proteins influence the uptake and distribution of elements in plants. Cd from soil and the atmosphere accumulates in plants through root and leaf absorption. Currently, several transporter proteins related to Cd and its chelates, such as ZIP and YSL, have been identified using genetic engineering and molecular biology techniques (Cao et al., 2018). In this experiment, Cd exposure upregulated genes encoding ACA, ZIP, MGT, SULTR, VIT, and YSL, while CSF treatment significantly downregulated these genes (**Figure 12**). This suggests that co-application of silicon and iron reduces Cd uptake in tomato seedlings by suppressing the expression of transporter protein genes.

#### 3.5.6 DEGs involving transcription factors

Transcription factors (TFs) are crucial for plant development, TFs intricately govern cellular processes and responses to external stimuli by modulating gene expression (Dhatterwal et al., 2024). Cd notably influenced the expression of genes encoding TFs in tomatoes, while the addition of Si and Fe was able to alleviate the effect of Cd on TFs. The effect of Si and Fe co-application was more significant (**Supplementary Figure S5**).

### 3.6 WGCNA analysis

**Figure 10:**
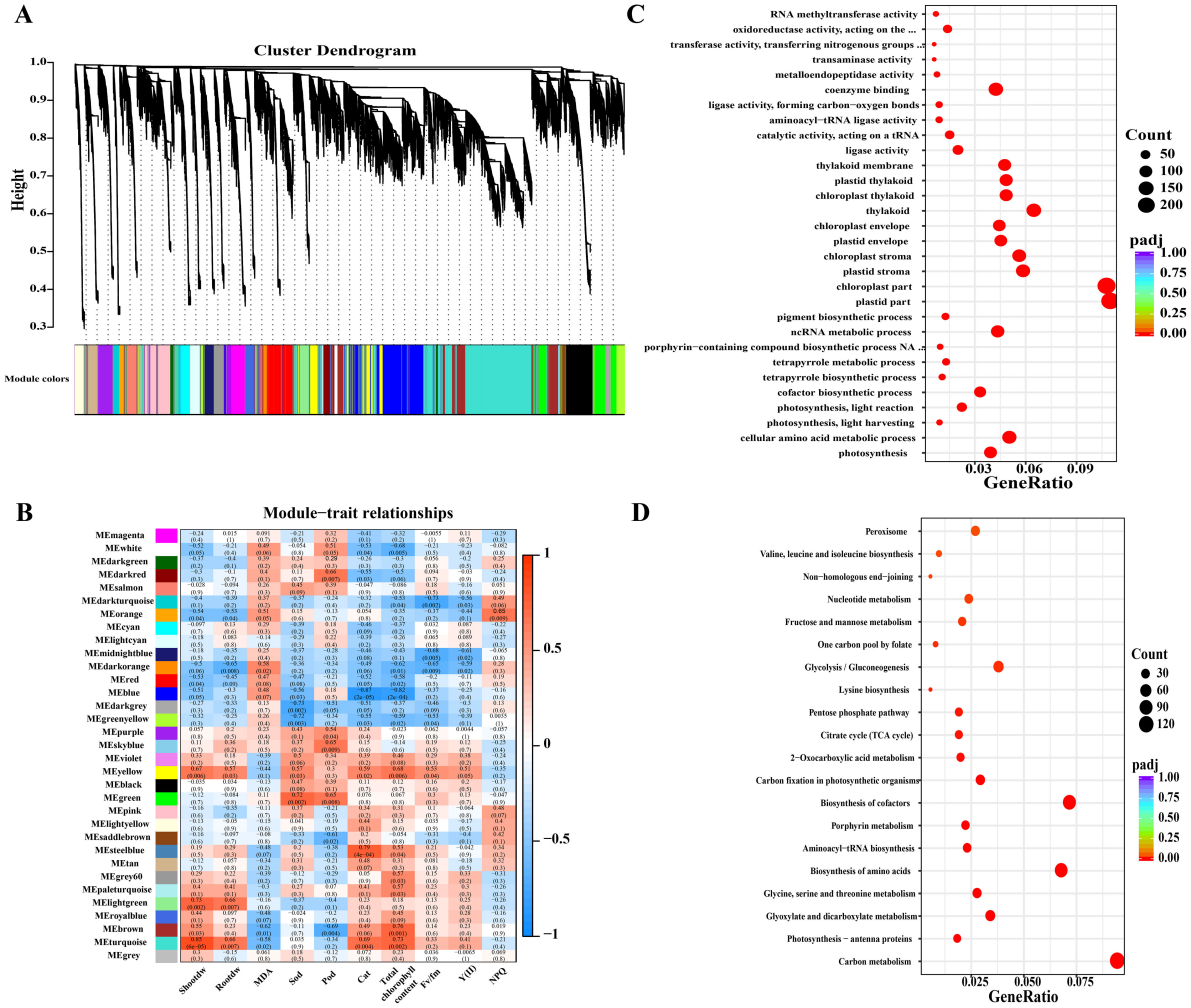
Genetic validation of tomato seedlings. **(A)** Cluster dendrogram, **(B)** module–trait relationship. Red panels indicate positive correlation, blue panels indicate negative correlation, and smaller numbers indicate lower *p*-values. **(C)** GO analysis and **(D)** KEGG analysis of tomato seedling leaves after treatment. **(C, D)** show GO and KEGG analyses of the turquoise module. The size of the bubbles indicates the number, and the color indicates the *p*-value: the redder the color, the lower the *p*-value.

Based on WGCNA, 33 unique modules were identified based on their correlation with the trait. Ten resistance indicators, including shoot dry weight, root dry weight, chlorophyll content, and the chlorophyll fluorescence index [Fv/Fm, Y(II), and NPQ], were used (**Figure 10B**). The findings indicate a positive correlation between genes in various modules and resistance indices. Turquoise, violet, and yellow modules were significantly correlated with shoot dry weight, root dry weight, CAT, and photosynthetic indices, including chlorophyll content, Fv/Fm, and Y(II). The turquoise module showed significant positive correlations with the photomagnetic, metabolic, and relative parameters. GO and KEGG analyses were performed following the analysis of the module genes. **Figures 10C, D** illustrate the GO and KEGG pathways associated with the turquoise module. The GO results indicated that most genes (*p* < 0.05) were enriched in photosynthesis reactions, cellular amino acid metabolism harvesting, photosynthesis–light reactions, plastids, chloroplasts, and coenzyme binding. In the KEGG analyses, the carbon metabolism, photosynthesis-hapten proteins, photosynthetic biocarbon fixation, citrate cycle, biosynthesis of amino acids and glyoxylate, and biosynthesis of cofactors were the most significantly enriched genes.

**Supplementary Figures S3A, B** show the results of GO and KEGG analyses of the other seven module genes. In GO analysis, genes were enriched in intracellular transport, intracellular protein transport, cellular macromolecule localization, protein localization, and protein transport. KEGG analysis revealed that the genes were enriched in ribosome plant hormone signal transduction and spliceosomes.

### 3.7 Visual analysis

**Figure 11:**
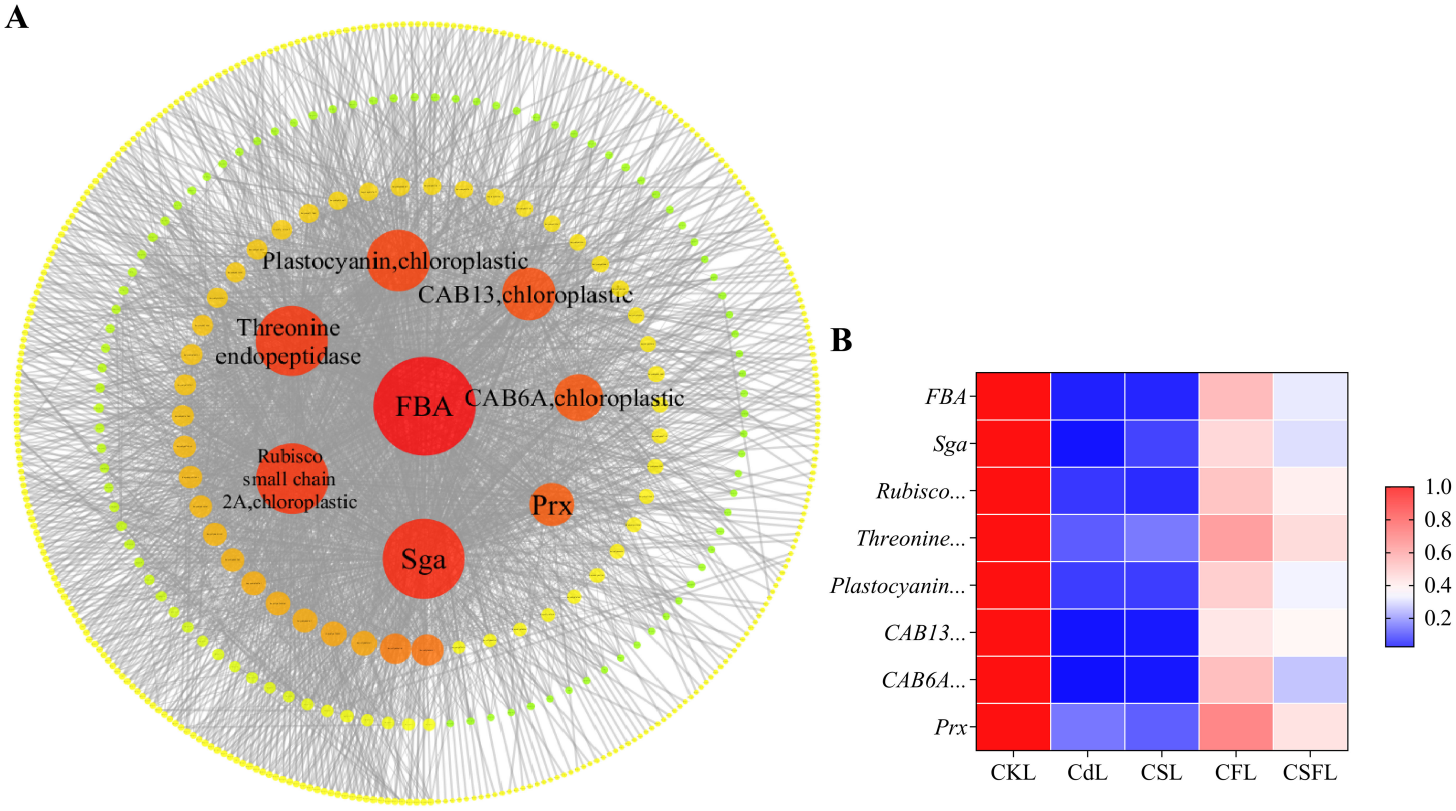
Genetic validation of tomato seedlings. **(A)** Visual analysis of the turquoise module. Bigger and redder bubbles mean higher scores. **(B)** Heat map of the eight main effector genes in tomato seedling leaves. Red and blue indicate upregulated and downregulated genes.

Since the number of genes in the turquoise module of WGCNA is even larger and the enriched pathway echoes the results of previous experiments, we further analyzed the turquoise module. In the turquoise module, eight hub genes were selected from the edges. They contain genes related to photosynthesis and stress tolerance. One of the selected genes was Solyc02g06234.3, which encodes a fructose-bisphosphate aldolase (FBA). Other genes included serine-glyoxylate aminotransferase; chlorophyll a-b binding protein 13, chloroplastic; RuBisCO small chain 2A, chloroplastic; threonine endopeptidase; plastocyanin, chloroplastic; peroxiredoxin (Prx); chlorophyll a-b binding protein 6A; and other chloroplastic genes (**Figure 11A**). **Figure 11B** shows the expression of these eight genes under different treatments compared to that in the CK, and all eight genes were downregulated in the Cd treatment. Compared to Cd, the expression of Sga and threonine endopeptidase was upregulated in CS, and all eight genes were significantly upregulated in CF and CSF.

**Figure 12:**
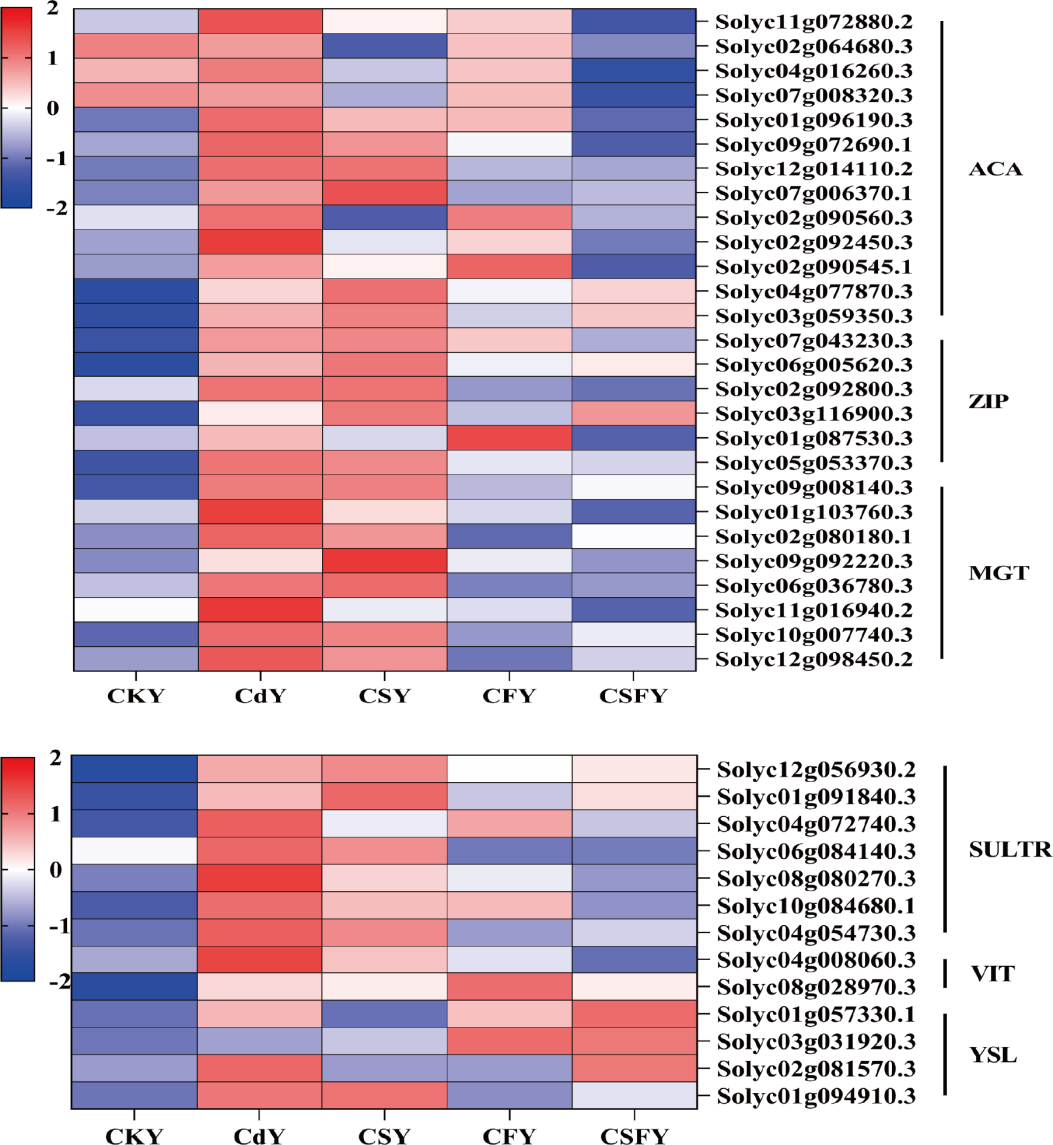
DEGs involving element transporters in tomato seedlings. ACA (Ca), ZIP (Zn/Fe), MGT (Mg), SULTR (SO42-), VIT and YSL (Fe). Color indicates the expression level: the redder the color, the higher the number of DEGs.

### 3.8 Model sketch

**Figure 13:**
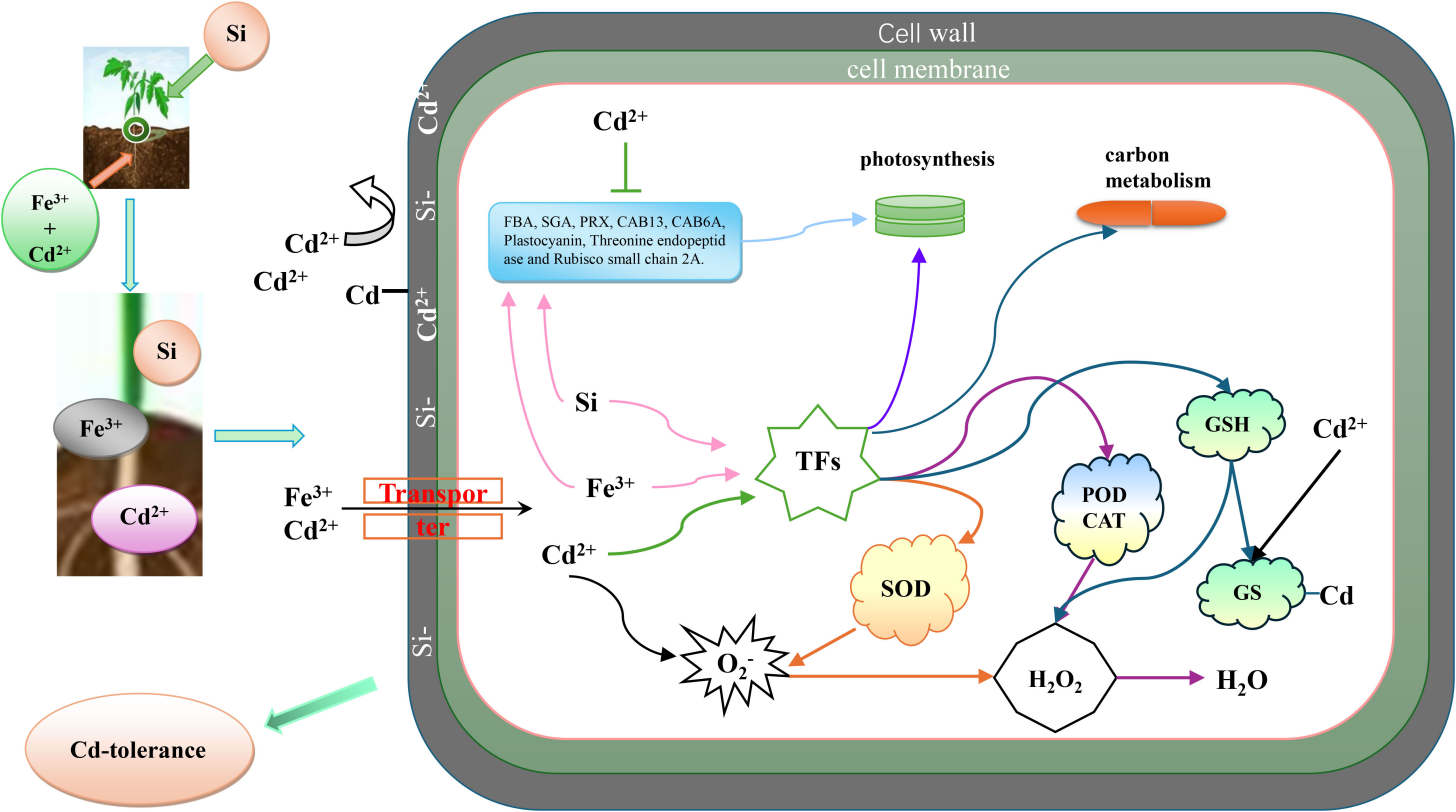
Model sketch.

The figure illustrates the application of combined silicon (Si) and iron (Fe) treatments to alleviate cadmium (Cd) stress in tomato seedlings. In this study, Si was applied to the leaves, while Fe was supplied to the roots. Silicon is incorporated into the cell walls, enhancing their strength and rigidity, which serves as the first line of defense by limiting Cd entry through adsorption and immobilization. Since plants lack specific transporters for Cd, Cd ions compete with essential elements such as Fe for uptake into cells. Upon entering the cells, Cd induces the excessive generation of reactive oxygen species, including superoxide anions (O^2-^), and alters the expression of certain TFs and genes, resulting in cellular damage. The combined application of Si and Fe effectively mitigates Cd-induced toxicity by reinforcing cell wall barriers, reducing Cd uptake, enhancing antioxidant enzyme activities, and stabilizing gene expression. These mechanisms collectively contribute to the protection of tomato seedlings against Cd stress (**Figure 13**).

## 4 Discussion

When plants take up excess Cd, it causes a variety of visible phytotoxic symptoms, such as inhibition of plant root growth and photosynthesis, alteration of protein metabolism and membrane function, and even death of the entire plant (Daud et al., 2009; Schutzendubel et al., 2001). Silicon (Si) is a highly effective element frequently used alongside other substances to alleviate plant stresses. For instance, Si combined with humic acid reduced Cd stress in strawberries (Dogan et al., 2022), while Si together with nitric oxide mitigated water stress in apricot and Myrobalan 29C rootstocks (Bakır et al., 2022; Bolat et al., 2022). In this study, Cd significantly inhibited the growth of tomato seedlings; however, the co-application of Si and iron (Fe) effectively alleviated Cd-induced damage, demonstrating their synergistic protective effect.

Si and Fe effectively reduce Cd uptake and translocation in tomato seedlings. Plants primarily absorb Cd through their roots, where most Cd^2+^ ions are immobilized as insoluble complexes within root tissues, limiting their movement. Only a small fraction of soluble Cd is transported to the aerial parts of the plant (Song et al., 2017; Lin et al., 2016). Both Si and Fe have been shown to inhibit Cd absorption and its translocation from roots to shoots (Lu et al., 2018; Chen et al., 2017). Consistent with these findings, our experiment demonstrated a significant reduction in Cd content in tomato seedlings subjected to Cd stress when treated with Si and Fe, highlighting their synergistic role in mitigating Cd accumulation. Genes used to encode ACA, ZIP, SULTR, MGT and VIT were significantly down-regulated (**Figure 12**).

Under cadmium (Cd) stress, silicon (Si) and iron (Fe) have been extensively reported to play crucial roles in protecting and enhancing the structural and functional integrity of the photosynthetic apparatus in plants. Silicon has been demonstrated to improve crop yields in maize and rice by alleviating Cd-induced damage to photosynthetic processes (Hussain et al., 2015; Mihaličová Malčovská et al., 2014), while iron mitigates Cd toxicity primarily by stabilizing the photosynthetic electron transport chain during the early stages of photosynthesis (Liu et al., 2020). In the present study, GO and KEGG pathway enrichment analyses consistently identified photosynthesis-related pathways as significantly affected. Furthermore, co-application of Si and Fe under Cd stress resulted in a marked increase in chlorophyll content and upregulation of genes encoding key photosynthetic components (**Figure 6**). These findings align with previous reports by Solti et al. (2016) and Yu et al. (2023). The comparatively limited effect of Si alone on photosynthetic performance under Cd stress may be attributable to differences in tomato cultivars or variations in treatment duration.

Extensive evidence indicates that silicon (Si) enhances plant antioxidant defenses by regulating the expression and activity of key antioxidant enzymes under abiotic stress conditions (Fang and Ma, 2005; Seyed Hajizadeh et al., 2023). Similarly, iron (Fe) mitigates stress in rice by upregulating antioxidant enzyme activities, thereby reducing oxidative damage (Masood et al., 2022). Moreover, GSH serves as an essential chelator of Cd, facilitating its detoxification and alleviating Cd-induced phytotoxicity (Delalande et al., 2010; Wang et al., 2021). In the present study, co-application of Si and Fe significantly enhanced the activities of key antioxidant enzymes in Cd-stressed tomato seedlings, which was accompanied by the transcriptional upregulation of genes encoding these enzymes (**Figures 8**, **9**, **Supplementary Figure S2**).

In this study, we performed transcriptome sequencing of tomato seedling leaves to elucidate the molecular mechanisms by which exogenous silicon (Si) and iron (Fe) alleviate Cd stress. GO and KEGG pathway analyses indicated that Si and Fe mitigate Cd-induced damage primarily through modulation of photosynthesis-related processes. WGCNA further revealed that photosynthesis is strongly associated with the turquoise module. Subsequent in-depth analysis of this module identified eight candidate genes that are likely to play pivotal roles in the synergistic alleviation of Cd stress by Si and Fe in tomato seedlings.

FBA is a key enzyme involved in glycolysis and gluconeogenesis, catalyzing the reversible cleavage of fructose-1,6-bisphosphate (FBP) into dihydroxyacetone phosphate (DHAP) and glyceraldehyde-3-phosphate (GA3P). Beyond its central metabolic role, FBA is integral to the Calvin cycle’s dark reactions, where it modulates photosynthetic efficiency in higher plants (Peme et al., 2021; Fushinobu et al., 2011; Jacques et al., 2018). Importantly, GA3P serves as a critical substrate for cellulose biosynthesis, contributing to cell wall formation—a structural barrier that facilitates Cd adsorption and sequestration, thereby mitigating Cd toxicity (Yang et al., 2019; Loix et al., 2017).

Sga, a signature enzyme localized within plant peroxisomes, is integral to the photorespiratory pathway, facilitating the recycling of metabolites essential for cellular homeostasis under stress conditions (But et al., 2019). Ribulose bisphosphate carboxylase small chain 2A, a chloroplast-localized subunit, is critical for the structural integrity and catalytic function of Rubisco, the primary enzyme responsible for CO_2_ fixation in photosynthesis (Spreitzer, 2003). Threonine endopeptidases mediate the proteolytic cleavage of internal peptide bonds, thereby regulating protein turnover and maintaining proteostasis. Plastocyanin, a soluble copper-containing protein in chloroplasts, acts as an electron donor to the P700 reaction center of photosystem I, while cytochrome f serves as an electron acceptor within the cytochrome b6f complex; both are essential components of the photosynthetic electron transport chain (Sigfridsson, 1998). Chlorophyll a-b binding proteins, including CAB13 and CAB6A, constitute a diverse family of light-harvesting complexes that capture and transfer solar energy to photosystem reaction centers, optimizing photosynthetic efficiency (Green et al., 1991). Peroxiredoxins, ubiquitous thiol-based peroxidases, play a vital role in scavenging reactive oxygen species and preserving cellular redox balance (Bhatt and Tripathi, 2011). The coordinated expression of these genes, which underpin photosynthetic performance and antioxidant defense, substantiates the protective effects of silicon (Si) and iron (Fe) supplementation in enhancing cadmium (Cd) tolerance in tomato seedlings.

## 5 Conclusion

The co-application of silicon (Si) and iron (Fe) emerges as a superior and synergistic strategy to mitigate cadmium (Cd) toxicity in tomato plants, surpassing the efficacy of individual treatments. This dual supplementation profoundly enhances seedling growth under Cd stress by significantly boosting antioxidant enzyme activities, optimizing photosynthetic capacity, and markedly reducing both MDA accumulation and Cd uptake. Comprehensive transcriptomic analyses demonstrate that Si and Fe co-treatment effectively reverses Cd-induced dysregulation of gene expression and TFs, thereby protecting the integrity of the photosynthetic machinery. Notably, eight candidate genes—FBA, Sga, ribulose bisphosphate carboxylase small chain 2A (RuBisCO small chain 2A), threonine endopeptidase, plastocyanin, chlorophyll a-b binding proteins CAB13 and CAB6A, and Prx—have been identified as pivotal mediators of this protective response. These genes orchestrate essential biological functions including carbon metabolism, protein turnover, electron transport, light harvesting, and reactive oxygen species detoxification, collectively fortifying photosynthetic efficiency and redox homeostasis. Together, these findings illuminate the multifaceted molecular framework through which Si and Fe synergistically enhance Cd tolerance, offering a promising avenue for sustainable crop production in heavy metal-contaminated soils.

## Data Availability

The original contributions presented in the study are included in the article/**Supplementary Material**. The raw sequence reads presented in the study are deposited in the NCBI repository, accession number PRJNA1245030. Further inquiries can be directed to the corresponding author(s).
