# Correction: Mitigation mechanism of silicon and iron co-application to cadmium toxicity in tomato seedlings by integrated transcriptomic and physiological correlation analysis

**DOI:** 10.3389/fpls.2025.1682501

**Published:** 2025-11-26

**Authors:** Xiaoting Zhou, Wenjie Wang, Deyang Ye, Xiaoru Liu, Chutong Peng, Yunxin Tang, Lihong Su, Shaobo Cheng, Kai Cao, Qiyuan Lei, Tonghua Pan, Zhongqun He

**Affiliations:** 1 College of Horticulture, Sichuan Agricultural University, Chengdu, Sichuan, China; 2 The Agriculture Ministry Key Laboratory of Agricultural Engineering in the Middle and Lower Reaches of Yangtze River, Institute of Agricultural Facilities and Equipment, Jiangsu Academy of Agricultural Sciences, Nanjing, Jiangsu, China; 3 School of Agricultural Engineering, Jiangsu University, Zhenjiang, Jiangsu, China

**Keywords:** cadmium, iron, silicon, tomato seedlings, toxicity

There was a mistake in the caption of **Figure 12** as published. **Figure 12** caption should not include “WGCNA analysis”. The corrected caption of **Figure 12** appears below.

**Figure 12:**
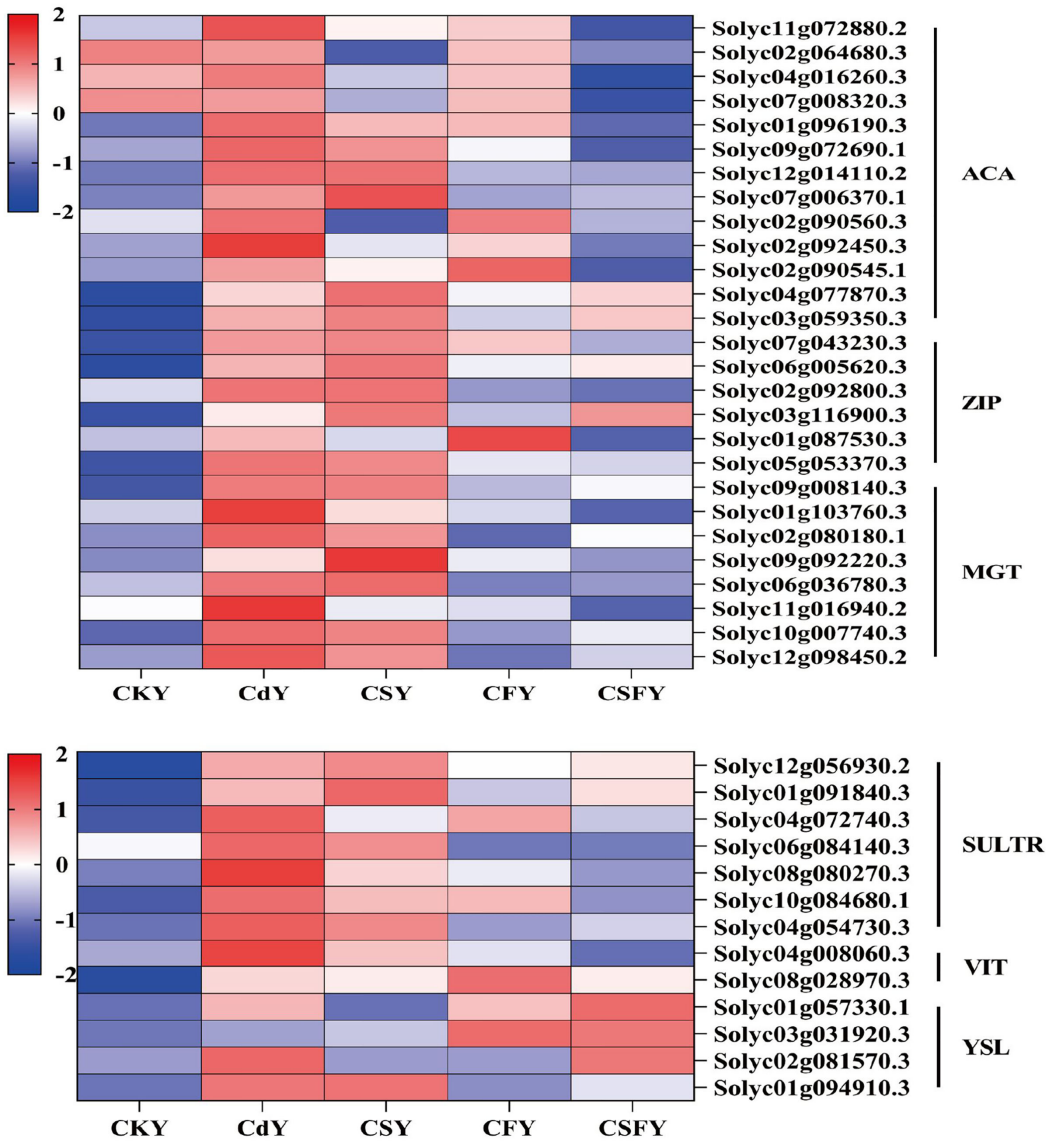
DEGs involving element transporters in tomato seedlings. ACA (Ca), ZIP (Zn/Fe), MGT (Mg), SULTR (SO42-), VIT and YSL (Fe). Color indicates the expression level: the redder the color, the higher the number of DEGs.

A correction has been made to the section 3.5.4 DEGs involving glutathione. The citation of “Figure 9” was missing from “Conversely, the expression of these genes showed an opposite trend in CS and CF compared to that in Cd and was significantly upregulated in CSF.” The correct phrase appears below:

“Conversely, the expression of these genes showed an opposite trend in CS and CF compared to that in Cd and was significantly upregulated in CSF (Figure 9).”

A correction has been made to the section 3.5.5 DEGs involving element transporters. The citation of **Figure 12** was missing from: “In this experiment, Cd exposure upregulated genes encoding ACA, ZIP, MGT, SULTR, VIT, and YSL, while CSF treatment significantly downregulated these genes.” The correct phrase appears below:

“In this experiment, Cd exposure upregulated genes encoding ACA, ZIP, MGT, SULTR, VIT, and YSL, while CSF treatment significantly downregulated these genes (**Figure 12**).”

In the published article, there was an error in *3.8 Model sketch* as published. Figure 13 and its citation disappeared from under the text “These mechanisms collectively contribute to the protection of tomato seedlings against Cd stress”. The corrected **Figure 13**, its citation and its caption appear below:

**Figure 13:**
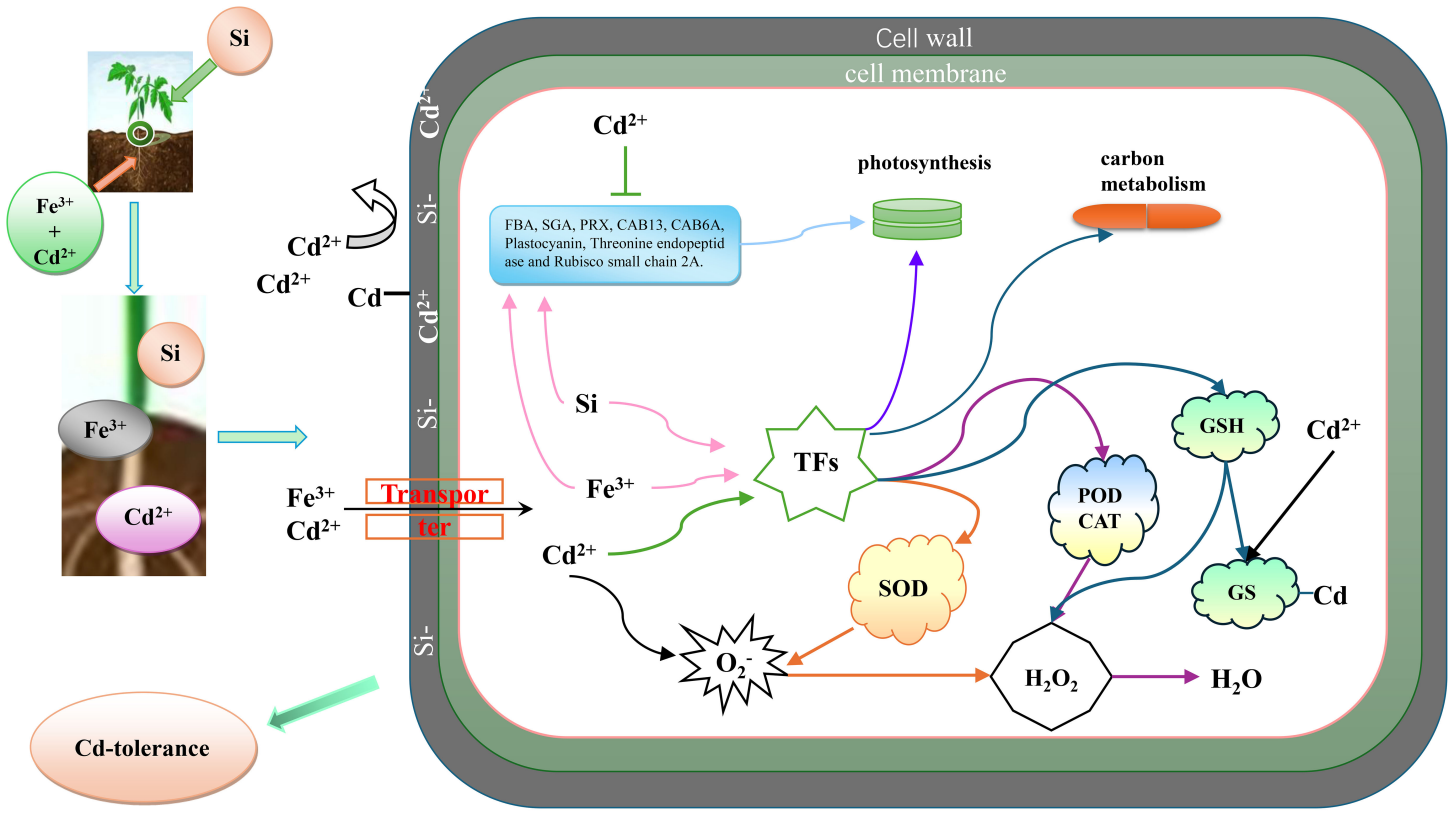
Model sketch.

“These mechanisms collectively contribute to the protection of tomato seedlings against Cd stress (**Figure 13**)”.

The original version of this article has been updated.

