# Supplementary material for: Mitigation mechanism of silicon and iron co-application to cadmium toxicity in tomato seedlings by integrated transcriptomic and physiological correlation analysis
Source: Front Plant Sci. 2025 Jun 30;16:1555618. doi: 10.3389/fpls.2025.1555618 (PMC12257105; doi:10.3389/fpls.2025.1555618)
Supplement: Supplementary file 1 [file Image1.pdf]

Supplement table:

Gene name	primer	primer sequence
Actin	upstream primer	GGGATGGAGAAGTTTGGTGGTGG
	downstream primer	CTTCGACCAAGGGATGGTGTAGC
Solyc04g015970.3	upstream primer	ACGCCATAGAAGCAGCAGGATC
	downstream primer	TGACTATCGCTCCATACCTTGACTG
Solyc08g080890.3	upstream primer	GAAGCATTAGCAGAAACGGAAGGAC
	downstream primer	CAACGGGTCAGAAACGGAAAGAAC
Solyc08g083060.3	upstream primer	TAGTTCCGCTTAGAAAGGTCTTGATAG
	downstream primer	ACAGCAGTTGCCATTCCAGTTG
Solyc03g034220.3	upstream primer	GGTCTCAAATCTTCAGCCACTTTCC
	downstream primer	CATGCAGCTAACTCTTCCACCATTG
Solyc01g109880.3	upstream primer	CGTCAAGTGGTACATCATCAGGTTC
	downstream primer	TGATCTTCTTGCCGATTCACGATTC
Solyc02g063150.3	upstream primer	ACACAGGTGTTGGCTGAGGTTC
	downstream primer	GAATCCTTCTGGCTTGTAGGCAATG
Solyc11g012700.2	upstream primer	GTCCAACCACCACCGCTCTC
	downstream primer	GCTACCTCCTCCACCGAACATC
Solyc02g085950.3	upstream primer	GTCCACTGCTTCTTTCCCTGTTTC
	downstream primer	AACTGACTCTTCCACCGTTGCTAG
HMA3	upstream primer	GCGATTACAGACCATGCCACAAC
	downstream primer	GCGAACAAACCTTACGACCACTG
Nramp6	upstream primer	GGGTTGTTACAGGAAAGCATCTAGC
	downstream primer	CAGGAATATCACACGCCACAATAGC
IRT1	upstream primer	CACGTTCTATTCCGGCCCTAAGC
	downstream primer	CCGTAGCCAGGATAATTCCAGCAG

Table. 1. Fluorescent quantitative gene primer information.

Supplement figure:

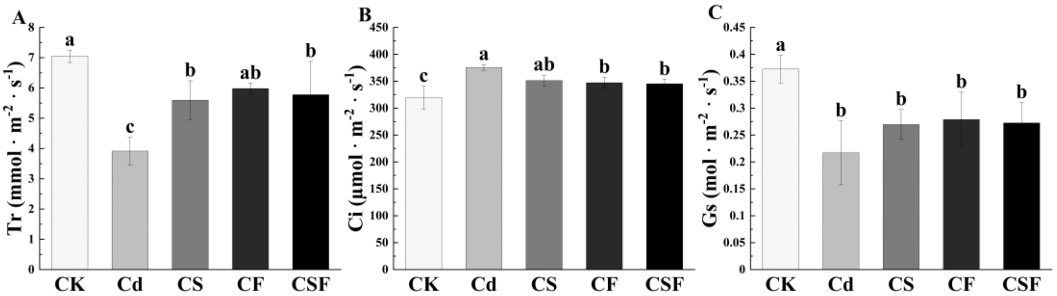

Figure. S1. Different treaments on Chlorophyll fluorescence parameters in tomato seedlings. (A) Tr, (B) Ci and (C) Gs. Different letters (a–c) on the bar plots indicate significant difference at $P < 0.05$ using one-way analysis of variance with Duncan's multiple-range test.

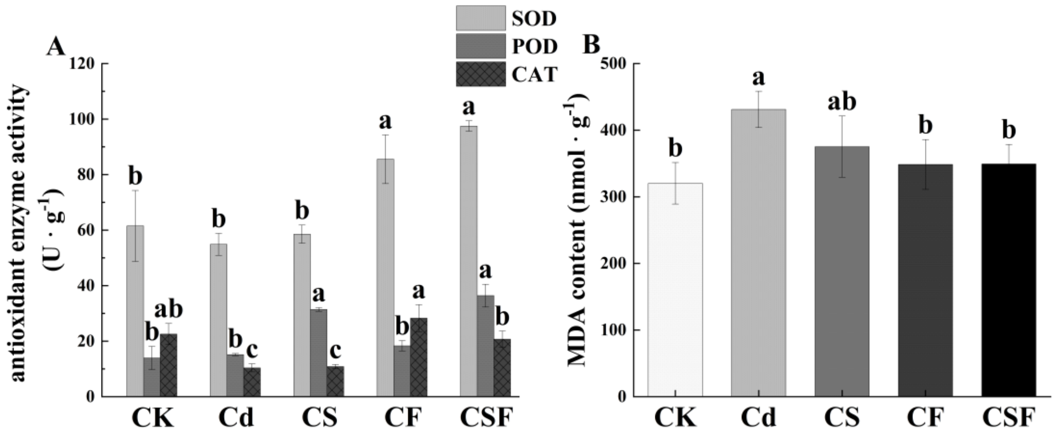

Figure. S2. Different treaments on antioxidant enzyme activity and MDA content in tomato seedings. (A) antioxidant enzyme activity, (B) MDA content. Different letters (a–c) on the bar plots indicate significant difference at $P < 0.05$ using one-way analysis of variance with Duncan's multiple-range test.

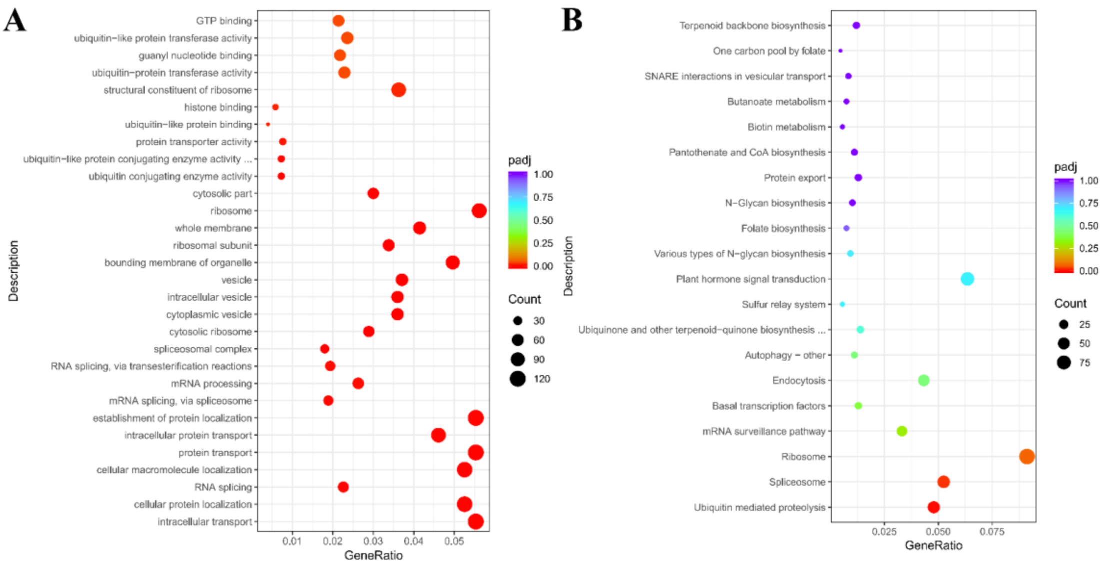

Figure. S3. (A) GO and (B) KEGG results from seven modules: black, purple, yellow, green, purple, sky-blue and dark. The size of the bubbles indicates the number and the color indicates the *P*-value: the redder the color, the lower the *P*-value.

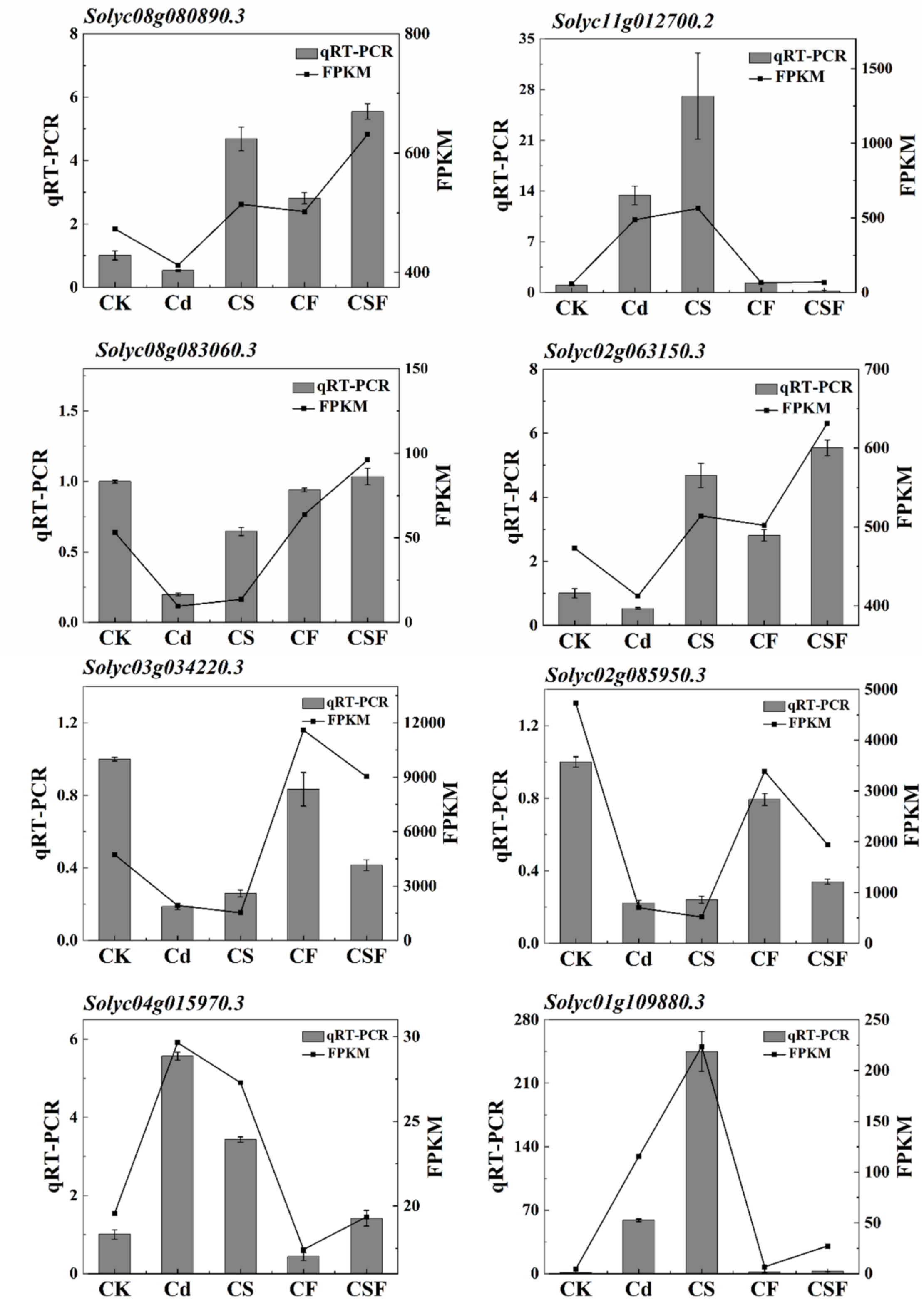

Figure. S4. Genetic validation in tomato seedlings. RT-qPCR validation of differentially expressed genes.

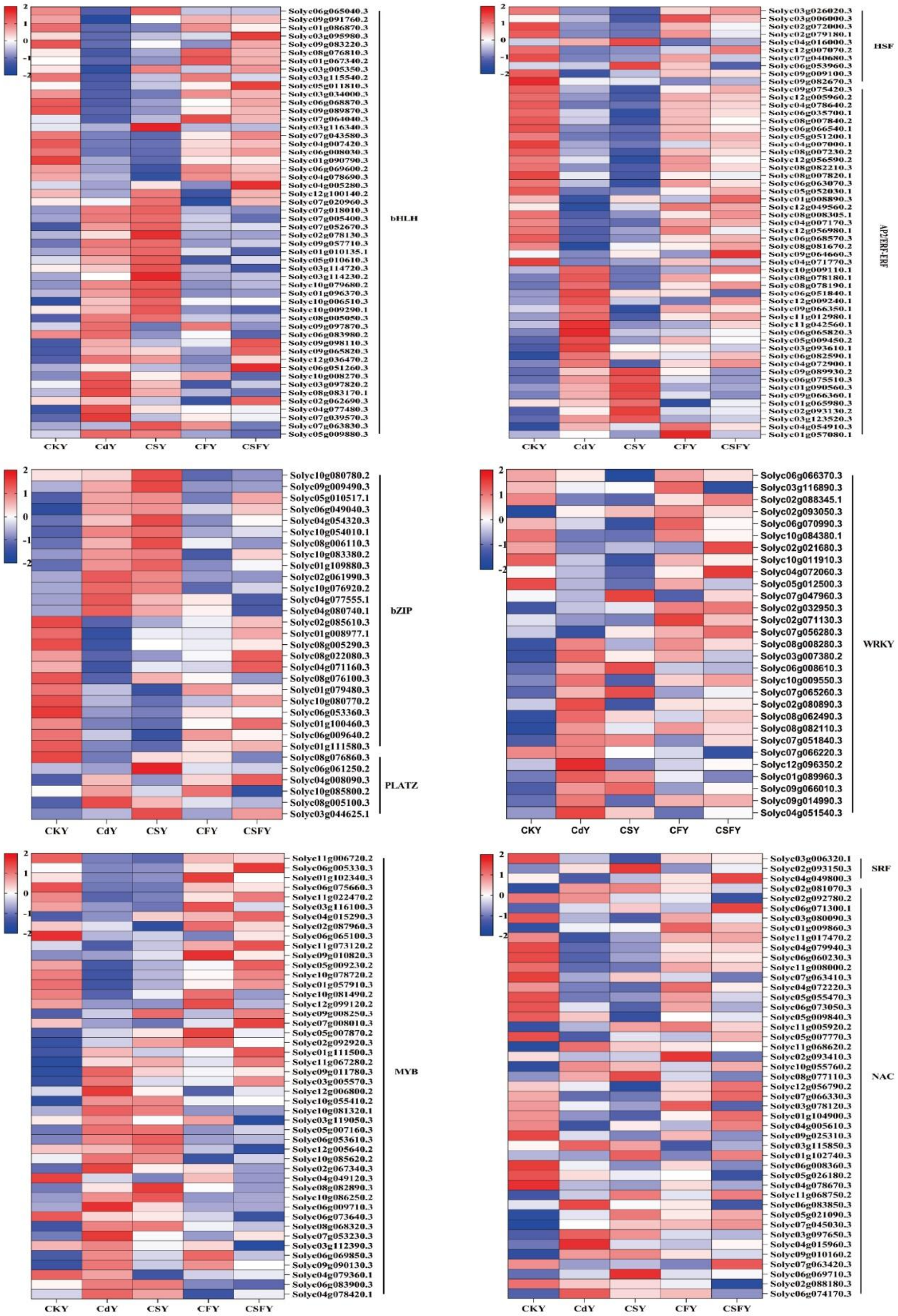

Figure. S5. DEGs involving TFs in tomato seedlings. The color indicates the expression level: the redder the color, the higher the number of DEGs.

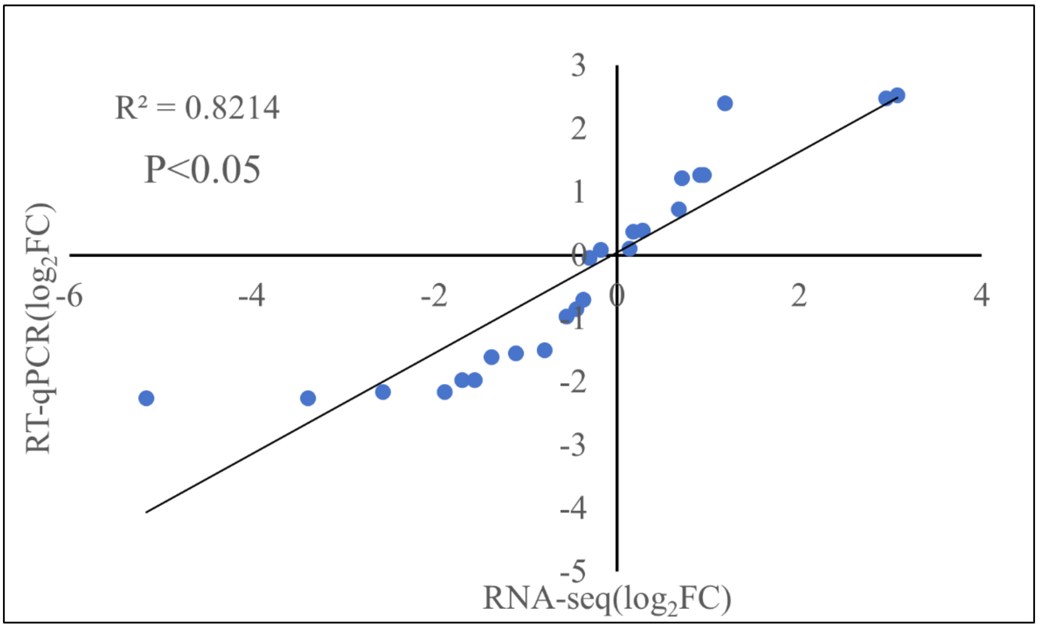

Figure. S6. Eight selected genes were validated using the RT-qPCR method comparatively, and linear correlation between RNA-seq and RT-qPCR data was presented with a $\log_2$ fold change transformation.

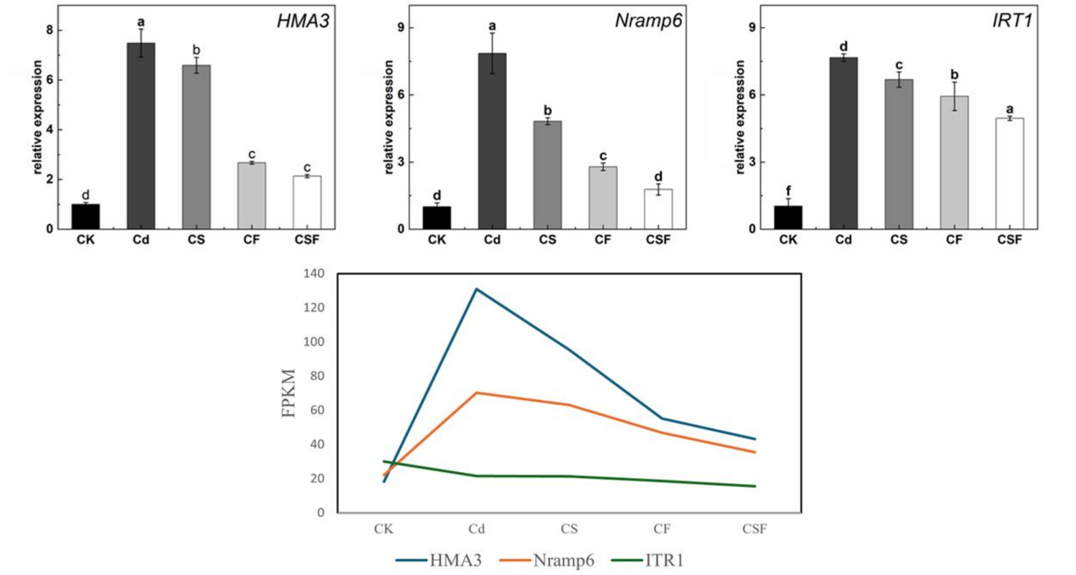

Figure. S7. Genetic validation in tomato seedlings. RT-qPCR validation of differentially expressed genes.
